# Expression of Glucose Transporters in the Prelaminar Region of the Optic-Nerve Head of the Pig as Determined by Immunolabeling and Tissue Culture

**DOI:** 10.1371/journal.pone.0128516

**Published:** 2015-06-01

**Authors:** F. Javier Carreras, Carlos J. Aranda, David Porcel, Francisco Rodriguez-Hurtado, Olga Martínez-Agustin, Antonio Zarzuelo

**Affiliations:** 1 Department of Surgery (Ophthalmology), Faculty of Medicine, University of Granada, Granada, Spain; 2 Department of Biochemistry and Molecular Biology II, Faculty of Pharmacy, University of Granada, Granada, Spain; 3 Center of Scientific Instrumentation, University of Granada, Granada, Spain; 4 Division of Ophthalmology, Licinio de la Fuente University Hospital, Granada, Spain; 5 Department of Biochemistry and Molecular Biology II, Faculty of Pharmacy, University of Granada, and Networked Biomedical Research Center for Hepatic and Digestive Diseases (CIBEREHD), Granada, Spain; 6 Department of Pharmacology, Faculty of Pharmacy, University of Granada, and Networked Biomedical Research Center for Hepatic and Digestive Diseases (CIBEREHD), Granada, Spain; University of Sydney, AUSTRALIA

## Abstract

**Background:**

To develop the use of cultured tissue of the prelaminar optic nerve of the pig to explore possible alterations of the astrocyte-axon metabolic pathways in glaucoma, we map the distribution of the glucose transporters GLUT1 and GLUT3 in fresh and cultured tissue.

**Methods:**

We monitor cell survival in cultures of the prelaminar optic-nerve tissue, measuring necrosis and apoptosis markers biochemically as well as morphologically, and establish the presence of the glucose transporters GLUT1 and GLUT3. We map the distribution of these transporters with immunolabeling in histological sections of the optic nerve using confocal and electronic transmission microscopy.

**Results:**

We find that the main death type in prelaminar culture is apoptosis. Caspase 7 staining reveals an increment in apoptosis from day 1 to day 4 and a reduction from day 4 to day 8. Western blotting for GLUT1 shows stability with increased culture time. CLSM micrographs locate GLUT1 in the columnar astrocytes and in the area of axonal bundles. Anti-GLUT3 predominantly labels axonal bundles. TEM immunolabeling with colloidal gold displays a very specific distribution of GLUT-1 in the membranes of vascular endothelial cells and in periaxonal astrocyte expansions. The GLUT-3 isoform is observed with TEM only in axons in the axonal bundles.

**Conclusions:**

Tissue culture is suitable for apoptosis-induction experiments. The results suggest that glucose is transported to the axonal cleft intracytoplasmically and delivered to the cleft by GLUT1 transporters. As monocarboxylate transporters have been reported in the prelaminar region of the optic-nerve head, this area is likely to use both lactate and glucose as energy sources.

## Introduction

The relative importance of the production and use of lactate and glucose in the brain remains a debated issue. Although glucose is still considered essential for the function of nervous system cells, it is not believed to be used directly by all neurons. [1–4] In general, glucose enters cells through specific facilitative transporters, termed glucose transporters (GLUTs), that vary depending on the cell type.[5] Once in the cell, glucose is phosphorylated by hexokinase (HK) to produce glucose-6-phosphate. [4] This compound is incorporated in different metabolic pathways, including glycogen synthesis, lactate-energy production via glycolysis or entry into the pentose phosphate pathway. To exit the cell, glucose-6 phosphate must be dephosphorylated by glucose-6-phosphatase (G6Pase). Both the expression of G6Pase, specifically G6PaseE, and glycogen storage have been shown to be present in astrocytes [6], which are the main energy reserve in the brain. Although it has been suggested that astrocytes have the ability to convert glycogen into glucose [6,7], it is not known under what conditions the astrocyte glycogen is cycled into glucose *in vivo*, and there is sketchy and still controversial evidence that confers a glucose-export capacity on astrocytes *per se*. [6]

In the gray matter, protoplasmic astrocytes surround blood vessels with prolongations, called end-feet that express glucose transporter isoform GLUT1on the membrane. [8,9] In astrocyte cultures, lactate export predominates under most conditions. The available evidence indicates that as a part of the symbiotic relationship between astrocytes and neurons, the former constitute an energy source supplying neurons with an alternative substrate, lactate, that might be needed during an energy crisis. [10–15] In fact, the activation of neuronal metabolism leads to increased lactate use as a metabolic substrate by neurons, and lactate production and export predominate under most conditions in astrocyte cultures. For example, monosaccharide deprivation and increased metabolic demand induce glycogen conversion into lactate in astrocytes in culture [10] as well as in organotypic culture of the optic nerve (ON). [11,12]

In the preliminary region of the ON head (OHN), which is part of the central nervous system (CNS) [16], the axons of the neurons are naked in most mammals (with the exception of the rabbit) and make contact with each other forming bundles. [17] Astrocytes in this tissue, as the only glial cells responsible for the maintenance function of the axons, take in glucose and, apart from satisfying their metabolic needs, transform it into lactate which can be exported through a monocarboxylate transporter (MCT1) to the intercellular cleft. Next, MCT2 in the axonal membrane of neurons can absorb the lactate. [18,19]. The distribution of MCT1 and 2 isoforms in the prelaminar region has recently been described as exclusively around the axonal bundles [20], confirming the hypothesis that considers the lactate shuttle to be an important source of axonal energy in the preliminary tissue of the ONH.

In contrast to MCTs, most GLUTs act passively, allowing the traffic of glucose by a concentration gradient. [21] Among its many isoforms, GLUT1 is known to be the glucose transporter for astrocytes [22] and GLUT3 for neurons. [22,23] GLUT1, the most abundant GLUT isoform in the brain, plays a major function in regulating ATP production through glucose metabolism in neurons and astroglia. [8] This transporter in the brain is localized in the capillary endothelium and in astrocytes, [24] serving endothelial cells by taking glucose from the blood stream and exporting it into the extracellular cleft between the endothelium and the astrocyte. [25] This same transporter (GLUT1) is used to internalize glucose into the astrocyte. GLUT3 is referred to as the neuron-specific glucose transporter [23] and is therefore the obvious candidate to consider for direct glucose intake by prelaminar-tissue axons.

The precise ultrastructural localization of GLUTs in the brain, including the ON, has proved especially difficult [26], and few studies address this subject (see Redzic, 2011 [27] for a review), with none centering on the prelaminar region of the ONH. Given recent findings identifying lactate as the main energy source in the brain, the distribution of GLUT1 and 3 in the ONH bears investigating, particularly because its prelaminar region is one of the purported damage sites in glaucomatous optic neuropathy, our target disease. The pathophysiology of glaucomatous optic neuropathy is still not well understood and, although apoptosis is known to be the mechanism of ganglion cell loss in glaucoma, whether the site of primary damage is the ganglion cell body or its axon remains under debate. [28]

Here, we use tissue culture of the prelaminar region of the ON of the domestic pig as a model to study astrocyte response to environmental changes. We examine the feasibility of using an *in toto* culture of the prelaminar region of the pig ONH to elucidate the possible role of glucose in the metabolic pathways in astrocytes, specifically those of the prelaminar region. To achieve this goal, we locate facilitative transporter molecules in the main tissue components, vascular endothelial cells, astrocytes, and neurons (axons). The relative contribution of glucose and lactate metabolism in the axons of the retinal ganglion cells (RGCs) at the level of the ON was deduced from the distribution of MCT and GLUT transporters.

## Methods

**Fig 1:**
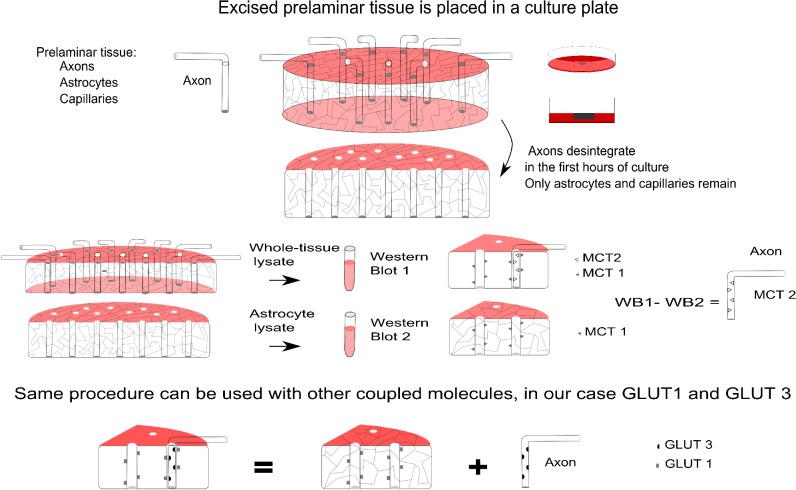
Schematic drawing of the experimental arrangement. The prelaminar region of the optic nerve head is excised and cultured in shallow medium to allow an adequate supply of oxygen to reach the tissue. As reported elsewhere, the axons are destroyed, leaving a matrix of astrocytes and capillaries that adapt to culture conditions. Microscopy (LM, TEM, and CLSM, including immunolabeling) is used to show that the tissue retains normal characteristics, including the important zonula adherens, and contains the glucose transport molecules GLUT1 and GLUT3. A comparison between the freshly excised and the cultured tissues by western blot shows the proportional quantities of the coupled molecules under study. This approach was followed in a previous report for MCTs and was performed here for GLUTs. (LM: light microscopy; TEM: transmission electron microscopy; CLSM: confocal laser scanning microscopy).

**Fig 2:**
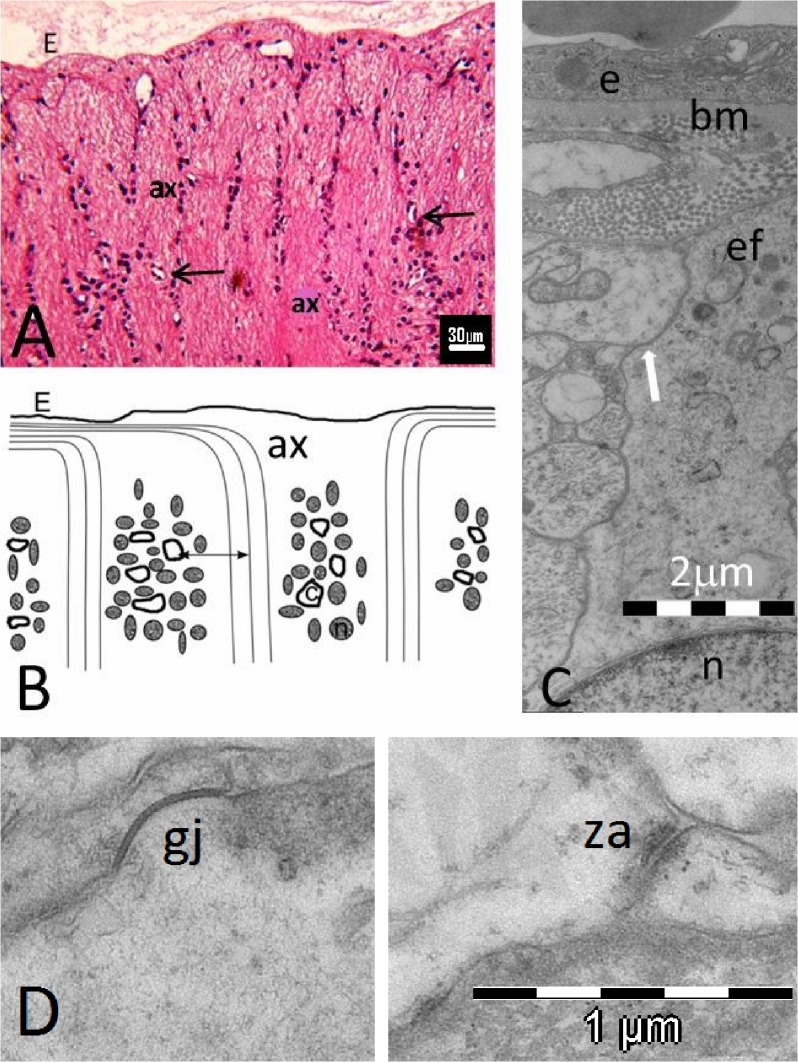
A: Sagittal section of the ONH of the pig. Hematoxylin and eosin. Magnification, 400x E: Elschnig’s membrane. The arrows point to capillaries. **A**: axonal bundles; c: capillary. **B**: Diagram of the sagittal section of the ONH emphasizing the arrangement of the astrocyte somas in columns alternating with axonal bundles. Only the nuclei of the astrocytes are represented. The capillaries are situated among the soma of the astrocytes in the columns. The two-headed arrow indicates the distance between capillaries and axons, a space filled with densely packed cell projections, as shown in **C**. ax: axon; c: capillary; n: nucleus of the astrocyte. **C**. TEM micrograph of a capillary of the astrocyte columns surrounded by astrocyte end-feet. The white arrow points to the narrow intercellular cleft between the densely packed cell projections. bm: basal membrane; e: vascular endothelial cell; ef: end-feet of astrocytes; n: nucleus of an astrocyte. **D.** TEM micrograph of astrocytic expansions in a 5-day culture of the prelaminar region. This image shows preservation of focal cell adhesion structures as gap junctions (GJ) and zonulae adherens (za). The magnification is given in the figure.

**Fig 1** is a schematic drawing of the experimental arrangement. Briefly, the prelaminar region of the ONH is excised and cultured in shallow medium. As reported elsewhere, axons are destroyed, leaving a matrix of astrocytes and capillaries that adapt to culture conditions. [20] A comparison between the freshly excised and the cultured tissues by microscopic and analytical methods renders visible the distribution and proportional quantities of the coupled molecules under study. This approach was used in a previous report with MCTs and is performed here with GLUTs. [20] **Fig 2A** gives a general idea of the tissue studied to express the main doubt that the work seeks to elucidate, as stated in the Introduction, and to stress the difficulties inherent in an intricate tissue organization that justify the following combination of different approaches: confocal laser scanning microscopy (CLSM), transmission electron microscopy (TEM) and western blotting of both fresh and cultured specimens. Any single approach to addressing the presence and distribution of GLUT molecules in the prelaminar tissue of the ONH would be insufficient on its own.

### Animals /Tissues

Eyes from six-month-old domestic pigs, collected at an abattoir soon after the animals were killed, were used directly for histological studies and for tissue culture of the prelaminar region of the optic nerve head (ONH). The time between the death of the animals and processing of the tissue averaged less than 3 h. The procedures followed in this research abided by the Declaration of Helsinki.

A total of 143 eyes were used for culturing and then processed for immunolabeling (CLSM and TEM, 48 pig eyes), JC-1 and TUNEL assays (32 pig eyes) and caspase 7 and GLUT detection by western blot, together with lactate dehydrogenase (LDH) in culture media (49 pig eyes). Additional pig eyes were used for simple TEM (6) and LM (4) studies. Additionally, 4 eyes from 4 normal Wistar rats (controls from a different experiment) were used as positive controls for GLU1 and GLUT3 expression.

Fresh eyes were dissected with blades and scissors. The optic nerve (ON) embedded in paraffin was prepared for light microscopy (LM) and confocal laser scanning microscopy (CLSM). The ON was embedded in resin for transmission electron microscopy (TEM), as detailed below. The eyes to be cultured were similarly processed but under sterile conditions.

### Antibodies and staining reagents

Both conventional and isotype-specific staining was performed. Specific anti-GLUT1 and anti-GLUT3 antibodies were used as primary antibodies, while secondary antibodies labeled with FITC were used for CLSM. Additionally, isotype-specific, FITC conjugated, secondary antibodies were used with the corresponding primaries in isotype-specific immunolabeling as a complementary control. The colocalization of GLUT-1 and GFAP was performed by double labeling at the CLSM. Meanwhile, 10-nm gold conjugates were used in labeling for TEM.

Information related to the use of antibodies, including the isotype controls, is summarized in Tables **1, 2 and 3**.

**Table 1:** List of conventional antibodies for Immunostaining.

Antibody	Laboratory	Species	Label	Final dilution	Proven reactivity
Anti-GLUT1	Santa Cruz Tech	goat IgG		1/100	mouse, rat, cow, human
Secondary antibody	Santa Cruz Tech	donkey anti-goat	FITC	1/100	
	Molecular Probes	rabbit anti-goat	Alexa Fluor 633	1/200	
	Sigma-Aldrich	goat anti-rabbit	Gold 10 nm	1/75	
Anti-GLUT3	Santa Cruz Tech	rabbit IgG		1/100	mouse, rat
Secondary antibody	Santa Cruz Tech	goat anti-rabbit	FITC	1/100	
	Santa Cruz Tech	goat anti-rabbit	Gold 10 nm	1/75	
Anti-GFAP	Molecular Probes	mouse	Alexa Fluor 488	25 μg/ml	human

Only the final dilutions used are listed.

**Table 2:** List of isotype antibodies for immunostaining.

Antibody	Laboratory	Isotype	Label	Final dilution	Proven reactivity
Anti-GLUT1	Pierce	mouse / IgG2a, kappa		1/200	human, rat
Mouse IgG2a Isotype Control	Pierce	mouse / IgG2a	FITC	1/400	Not Applicable
Anti-GLUT3	Pierce	rabbit / IgG		1/200	mouse (Ms), Rat (Rt)

Only the final dilutions used are listed.

**Table 3:** List of antibodies for western blot.

Antibody	Laboratory	Species	Label	Final dilution	Proven reactivity
Anti-GLUT1	Abcam	mouse		1/400	mouse, rat, cow, human
Anti-GLUT3	Abcam	mouse		1/400	mouse, rat, cow, human
Secondary antib.	Sigma	goat	HRP	1/1000	
Anti-Caspase 7	Abcam	mouse		1/1000	mouse, rat, sheep, rabbit, hamster, dog, human, pig, monkey
Secondary antib.	Sigma	rabbit	HRP	1/1000	

Only the final dilutions used are listed.

### Tissue-culture technique

Explants, 100 to 300 μm thick, of the dissected prelaminar region of the ONH of recently enucleated pig eyes were cultured *in vitro* following the method described by Stoppini et al. (1991) [29], as adapted by Carreras et al. (2011) [20]. Briefly, the optic discs were dissected with a surgical blade in a sterile environment. After the scission, the tissues were soaked twice in PBS supplemented with 100 mg/L streptomycin, 100,000 U/L penicillin, and 2.5 mg/L amphotericin B. Then, the pieces were maintained in 6-well plates with 1 ml of Dulbecco’s modified Eagle’s medium supplemented with 10% fetal bovine serum, 2 mM L-glutamine, 100 mg/L streptomycin, 100,000 U/L penicillin, and 2.5 mg/L amphotericin B in a humidified 5% CO2 atmosphere at 37°C. The medium was changed every 24 h. The rationale for this technique is to maintain a disc of tissue covered by a thin layer of medium to allow for gas exchange. The explants were removed at days 2, 4, 6, and 8 for apoptosis assays and for western blot analysis and at days 3, 5, and 8 for TEM and CLSM studies.

The CLSM samples were fixed in glyoxal (Shandon Lipshaw, Pittsburgh, PA, USA) for 24 h and then processed for paraffin embedding. Sections of the embedded tissue were labeled with fluorescent antibodies against GLUT1 and GLUT3. The nuclei were counterstained with DAPI (Vectashield, Vector Laboratories, CA, USA).

### Confocal laser scanning microscopy (CLSM)

General procedure: The tissues were fixed in glyoxal and then processed for paraffin embedding. Five-micron thick sections were cut and mounted on polylysine-coated plates. Serial sections were washed in PBS after deparaffinization with xylene and graded alcohol. The sections were incubated in pre-diluted block serum for 1 h and then incubated with the primary antibody overnight in PBS at 4°C. Control sections that did not receive the primary antibody were maintained in PBS.

We performed several rounds of immunolabeling using both conventional and anti-idiotypic secondary antibodies. Double-labeling was performed by simultaneous incubation with both primary antibodies overnight. Nuclear counterstaining for CLSM was performed with mounting media using DAPI. The sections were examined and photographed using a Leica SP5 confocal laser microscope.

### Techniques for apoptosis in tissue culture

DAPI: To assess the pyknotic nuclear changes detected in apoptosis, the cells were stained with DAPI. Sections of paraffin-embedded samples were processed in the same manner as for those for CLSM but were stained only with DAPI.TUNEL: For the assessment of apoptotic cell death, the TUNEL assay was performed according to the manufacturer’s protocol and using DAPI as a nuclear contrast (Click-iT TUNEL Alexa Fluor Imaging Assay from Life Technologies Europe BV, Bleiswijk, Netherlands). The sections were permeabilized with 0.25% (v/v) Triton X-100 in PBS for 1 h at room temperature and rinsed PBS. The sections were processed in the same manner as those for CLSM. The fluorescence staining was photographed using a confocal laser-scanning microscope. JC1, MITOCHONDRIAL IMAGING: Astrocyte mitochondria were labeled with 5,5',6,6'-tetrachloro-1,1',3,3'-tetraethyl-benzimidazolocarbocyanine (JC-1, Life Technologies Europe BV, Bleiswijk, Netherlands). JC-1, a Nernstian dye, is a marker for the mitochondrial membrane potential. It is a cationic carbocyanine dye that accumulates in mitochondria. At low concentrations, the dye is present as a monomer and yields green fluorescence. At higher concentrations, the dye forms J-aggregates with a broad excitation spectrum, and emission varies from red to orange and yellow, depending on the concentration. We used a modification of the technique described by Simpson and Russell, 1998. [30] Cultured discs of the prelaminar region were used directly for incubation with JC-1 10 mM in DMSO. The incubation period ranged from 1 to 4 h at room temperature; DAPI was used for nuclear contrast. The discs were rinsed in PBS and deposited in well slides, covered with a glass cover slip and sealed, and immediately examined with CLSM. Fluorescent mitochondria were monitored at a magnification ranging from 1000 to 2000 x. Fluorescence images were acquired in both the green (488 nm excitation, 500–550 nm emission) and orange-red (545 nm excitation, 600–650 nm emission) spectral ranges, spanning the fluorescence emission bands of JC-1. Variations in mitochondrial size, together with changes in the membrane potential, were used as signs of apoptosis.

### Transmission electron microscopy (TEM)

The prelaminar region of the ONHs was sectioned with a Parker blade, and the sections were deposited in fixative. For simple TEM, 2% glutaraldehyde and 2% formaldehyde in a cacodylate buffer were used as a fixative. After fixation, the specimens were rinsed several times with a buffer solution followed by post-fixation with 1% osmium tetroxide for 1 h. After being rinsed again with PBS buffer for 15 min, the tissue specimens were dehydrated with a series of graded ethyl alcohols ranging from 70% to 100%. The samples were embedded in epoxy resin. The resin blocks were initially thick-sectioned at 1–2 μm with glass knives using an Ultramicrotome Leica Ultracut S and stained with Toluidine blue. These sections were used as a reference to trim blocks for thin sectioning. The appropriate blocks were then sectioned with a diamond knife at 70–90 nm, and the ultrathin sections were placed on copper mesh grids. After drying on filter paper, the sections were stained with uranyl acetate and lead citrate for contrast. After drying, the grids were viewed and photographed using a Zeiss Libra 120Plus electron microscope.

For immunochemical procedures, the tissue was fixed with 2% paraformaldehyde and 0.2% glutaraldehyde in PBS buffer. For better preservation of antigenicity, no osmium tetroxide was used. London White embedding resin was used. Some nickel grids were incubated with the secondary antibody as a control, but they were not incubated with the primary antibody. Others grids were incubated in block with BSA for 10 min. The sections were then incubated overnight with the primary antibody (see Table 1). The same primary monoclonal antibodies were used for TEM and CLSM. The washed grids were then incubated with the secondary antibody labeled with 10 nm Nanogold particles. After drying on filter paper, the sections were stained with uranyl acetate for contrast and examined with TEM. No lead citrate was used to avoid masking the gold marker.

### Lactate dehydrogenase (LDH) assay in tissue culture medium

Cellular toxicity was measured as the release of LDH [31]. The cells were cultured in the conditions described above, and LDH activity in the supernatant was spectrophotometrically measured using sodium pyruvate (25 mmol/L) as a substrate in 50 mmol/L sodium phosphate buffer (pH 7.5).

### Western blot analysis for GLUT1 and GLUT3 and Caspase 7 expression in prelaminar optic nerve

Emulsified cultures of prelaminar ONH were processed for western blotting for GLUT1, GLUT3 and a control protein (α-actin). An initial western blot analysis was performed in 5 fresh samples to determine the detectability of GLUT1 and GLUT3 in the prelaminar ON of the pig. The cultured samples were allotted to account for cell loss to apoptosis. Two samples were collected on day 2 of culture, 4 on day 4, 6 on day 6, and 10 on day 8. This experiment was repeated twice, for a total of 44 cultured eyes. All of the samples were subjected to the same analysis to study variations over the culture period.

Samples of the prelaminar region of the ONH, control or cultured, were homogenized in buffer (20 mmol/L Tris-HCl, 5 mmol/L EDTA, pH 7.5, 10 mmol/L sodium pyrophosphate, 100 mmol/L sodium fluoride, 1% Igepal, 2 mmol/L sodium orthovanadate, 1 mmol/L phenylmethylsulfonyl fluoride, 10 μg/ml aprotinin and 10 μg/ml leupeptin). The homogenates were centrifuged at 12,000 ×*g* for 10 min at 4°C. Protein concentrations in homogenates were measured using the bicinchoninic acid protein assay. Thirty micrograms of protein from each sample were subjected to 10% SDS-PAGE and were electrophoretically transferred to nitrocellulose membranes overnight. The mouse anti-GLUT1 and anti-GLUT3 antibodies (Abcam Lab.) were used at manufacturer-recommended dilutions. Information related to the use of antibodies for WB is summarized in **Table 3**.

After detection, the films were scanned, and densitometric analysis was performed on the scanned images using Scion Image-Release Beta 4.02 software (http://www.scioncorp.com).

A quantitative comparison between a cultured and a control sample was calculated in the following manner: relative values were expressed as means, indicating the values from densitometric analysis normalized to β-actin staining, relative to control measurements, which were assigned a value of 100. Equal loading was checked routinely by reversible Ponceau staining. [32]

### Statistical analysis

In all of the experiments, samples were run in triplicate, and the results are expressed as means with their standard errors. Differences among the means were tested for statistical significance by one-way ANOVA and a posteriori Fisher least significant difference tests on preselected pairs. All of the analyses were carried out with the SigmaStat 3.5 program (Jandel Corporation). Differences were considered significant at P< 0.05.

### General remarks on the methods

Apoptosis, or programmed cell death, can be recognized by characteristic morphological, biochemical, and molecular changes that have been broadly grouped into three stages: early, intermediate, and late. The procedures used in our study to detect apoptosis are based either on morphology (CLSM) and are essentially qualitative (i.e., show the presence or absence of apoptosis in any location), or they are analytical (WB) (i.e., allow the quantification of an intermediate molecule that increases in the apoptotic process and is unable to locate precisely where in the tissue it takes place). The two procedures are complementary. **Table 4** summarizes the methods used to detect apoptosis at different stages, indicating its qualitative or quantitative character in the present use and the phase of apoptosis that each method detects. JC-1, DAPI, and TUNEL staining are qualitative tools to show different phases of apoptosis. WB of Caspase 7, an executioner member of the caspase family, is used to quantify mid-stage apoptosis and its variations over culture time.

**Table 4:** List of procedures used to detect apoptosis.

Method	Qualitative/Quantitative	Type	Phase of apoptosis
JC-1	Qualitative	CLSM Microscopy	Early
WB of Caspase 7	Quantitative	Analytical technique	Intermediate
TUNEL	Qualitative	CLSM Microscopy	Intermediate and late
DAPI DNA staining	Qualitative	CLSM Microscopy	Late

Regarding immunostaining, our method was to use the control section without a primary antibody to set the acquisition parameters of the microscope. We chose this method so that there was no background and to make the image completely dark. Any fluorescence observed when exploring the specimen with primary and secondary antibodies was considered positive when the anatomical distribution of the labeling was specific. The background was eliminated because the parameters were identical to those used for the control section. This method enabled a qualitative evaluation of the results, which were classified as negative or positive (weak, moderate or intense).

## Results

### Results of Tissue Culture

**Fig 3:**
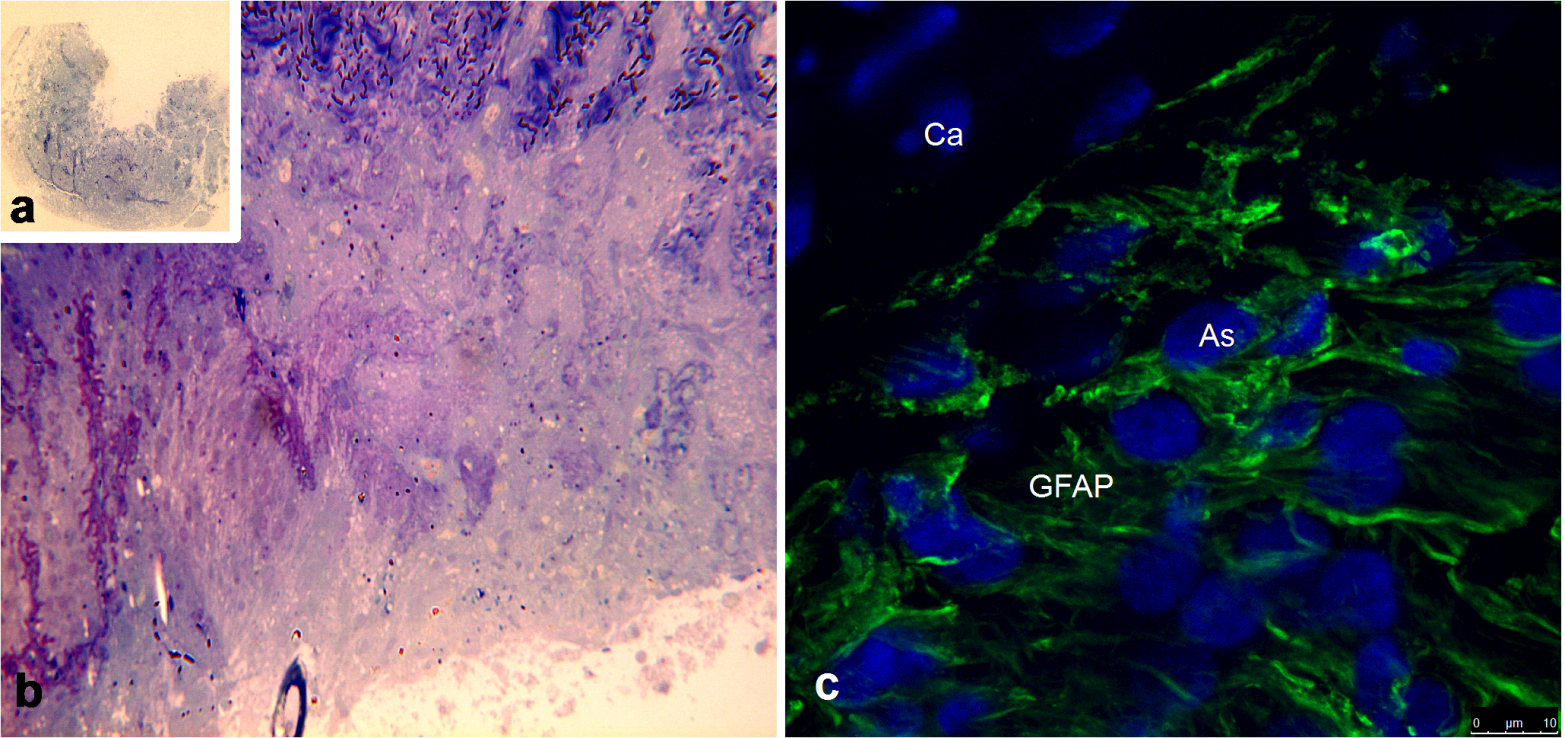
Explant of the prelaminar region of the optic nerve viewed via LM and CLSM. As explants are thinner than 300 microns, the penetration of culture medium is sufficient for the survival of the explant for more than a week. **A**) LM photography of an original 2 x magnification of an explant after 5 days of culture; toluidine blue staining of semithin section in epoxy resin. **B**) An explant after 6 days of culture at 40x; toluidine blue staining of semithin section in epoxy resin. **C)** CLSM micrograph of a prelaminar explant after 8 days of culture labeled for GFAP. GFAP is abundant, indicating the preservation of the main astrocytic characteristics, including rounded nuclei with a healthy shape stained with DAPI. A GFAP-negative capillary is present in the upper left corner and is delimited by astrocytic expansions.

**Fig 3** shows some characteristics of the explant, such as typical size and morphology of the cellular components on different culture days. Fig 3C shows good preservation of GFAP and the nuclear shape of astrocytes.

**Fig 4:**
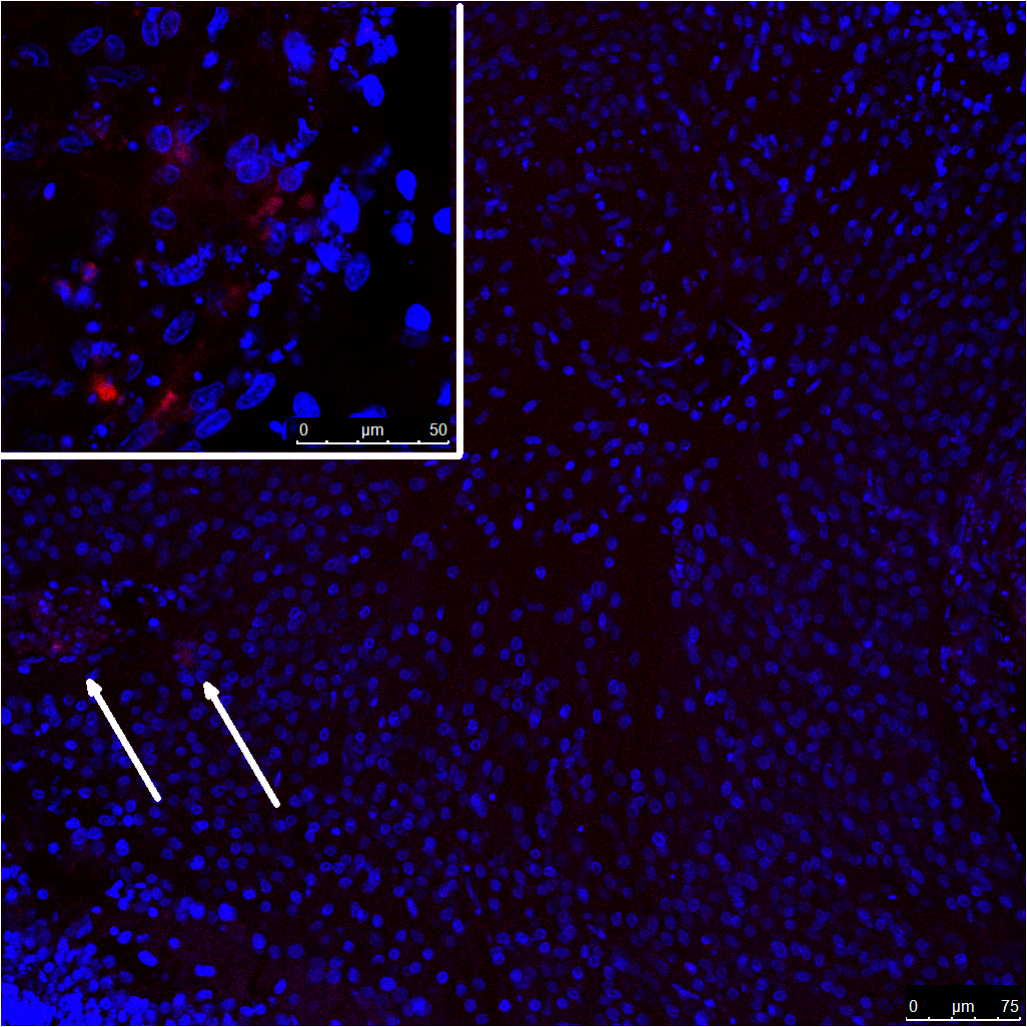
CLSM. TUNEL staining (red fluorescence) in a prelaminar tissue disc after 8 days of culture. After disintegration of the axons, the columnar arrangement of astrocytes is lost, and the tissue adopts a rounded shape formed by the grouping of the surviving cells. TUNEL staining is reduced to some peripheral areas of the explant (arrows). **Insert:** Some areas of TUNEL positive staining persist in nuclei after 8 days of culture. The magnification is given in the figures.

1. **Morphology, Apoptosis.** DAPI: Areas of cell destruction were noticeable over successive days of culture, beginning on day 1. Karyopyknosis (or nuclear shrinkage) and karyorrhexis (or nuclear fragmentation) were easily identified with DAPI in substantial areas of the tissue that expanded during the first few days of culture; these areas began to retract from day 4 onwards. The population of surviving astrocytes tended to coalesce, forming compact spherules with variable signs of nuclear involvement, from normal to pyknotic. (**Fig 4**)

TUNEL: In areas of karyopyknosis and karyorrhexis, positive red TUNEL staining was frequent, indicating that the main process of cell death was apoptosis. As with nuclear condensation and fragmentation, which was identified with DAPI in substantial areas of the tissue, the TUNEL effect increased during the first few days of culture and began to recede from day 4 onwards up to day 8. At this point, very few red staining areas were discernible. (**Fig 4**)

**Fig 5:**
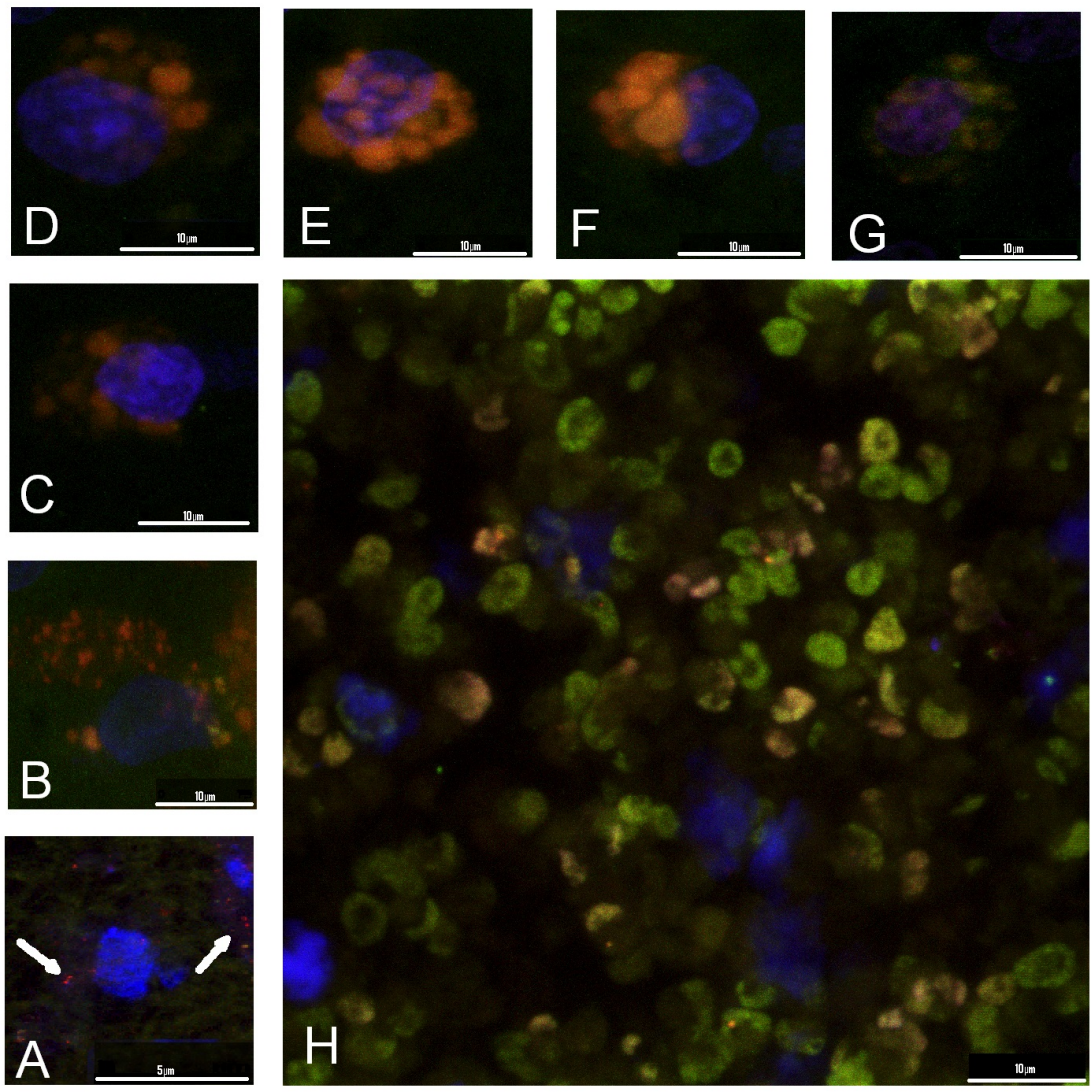
A to H. Tissue culture of the prelaminar region, JC-1 staining. A sequence showing the transformation from normal mitochondria into vesicular, inflated mitochondria. Healthy mitochondria are small compared to the nucleus (blue fluorescence). The normal metabolic activity of the mitochondrial membrane concentrates the dye (JC-1) on the inside and favors the formation of J-aggregates that fluoresce in bright red (A). As the ionic activity of the membrane decreases, the mitochondrion swells, the dye is less concentrated, and the hue changes from red to orange and finally to green (**B** to **H**) before mitochondrial disruption. This effect indicates mitochondrial-activated apoptosis as a main path for cell loss in prelaminar tissue culture. Here, the inflation of the mitochondria results from the decay of ionic pump function, which manifests as an increase in mitochondrial size and dilution of the dye from bright red to orange, yellow and green when increasingly diluted.

JC-1: There very few instances in which red mitochondria were clearly seen in cultured tissue. Red mitochondria are the result of the concentration of the dye in normally functioning cells. In our preparations, the characteristics of the cytoplasm in cells with red mitochondria suggested that the membrane of the cell was not intact. In our preparations, most of the astrocytes with normal nuclei did not accept the dye, and the mitochondria of normal cells were not stained. As in the case of TUNEL, substantial areas of the tissue showed stained mitochondria of increased size. Different concentration levels of the dye were observed as hues that varied from red to yellow and green, indicating a decaying membrane potential. According to subjective estimates, the affected areas reached their maximum extent around day 4 and shrank from day 4 to day 8. Apoptosis typically develops in less than 24 hours (Lemasters, 1998), and a culture of more than one day will show cells in different stages of apoptosis. (**Fig 5**)

**Fig 6:**
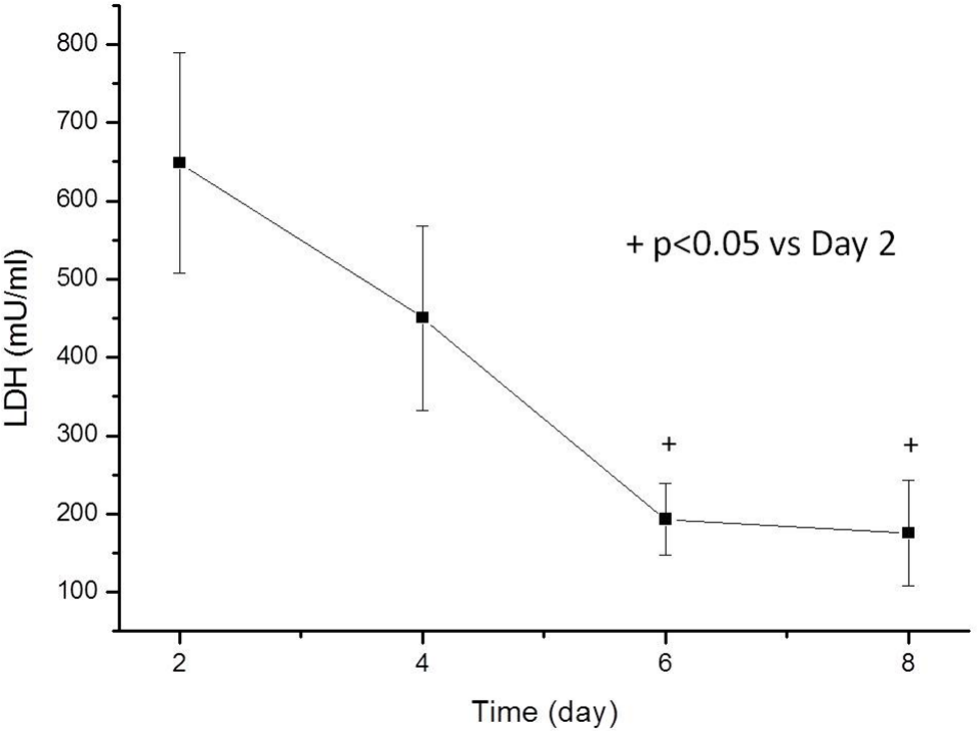
The levels of LDH in culture. The levels decrease from the first day of culture and stabilize at approximately day 6. Necrosis in the tissue is caused by the sectioning and manipulation process and is induced minimally or not at all by the culture conditions. The values are the means, with the standard errors represented by vertical bars. *The mean values were significantly different from those of the Day 2 group (P < 0.05).

**2. Biochemistry, necrosis.** LDH, in culture media: The amount LDH diminished from the first day of culture, reached a minimum around day 6, and stabilized from thereon. (**Fig 6**)

**Fig 7:**
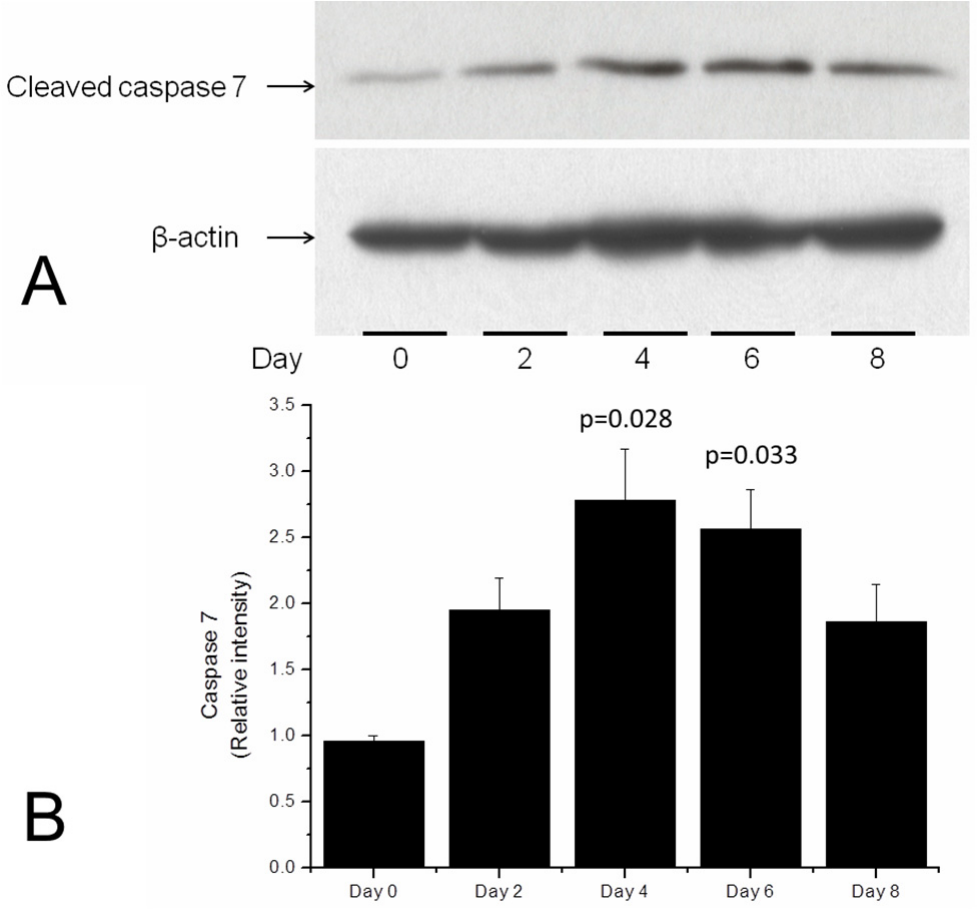
Western blot and graph of cleaved (active) caspase 7 levels in cultured discs of the prelaminar optic nerve head. A) Well-formed, compact bands of cleaved caspase 7 compared to the reference protein (β-actin). B) The quantification bars of three experiments. The number of discs harvested increased with culture time to compensate for cell loss due to apoptosis. Compared to samples of fresh tissue (Day 0), apoptotic activity as measured cleaved caspase levels increases following the first day of culture. After day 4, the number of cells undergoing apoptosis begins to decrease, indicating the stabilization of the culture. The values are the means, with the standard errors being represented by vertical bars.

**3. Biochemistry, Apoptosis.** Caspase 7 In Cultured Tissue: Coincidently with morphological studies, levels of cleaved caspase 7, which indicates active apoptosis, increased in the first days of culture, peaking on day 4. The levels began to fall from day 4 onwards to day 8, when levels of active caspase were similar to those observed on day 2. (**Fig 7**)

**Fig 8:**
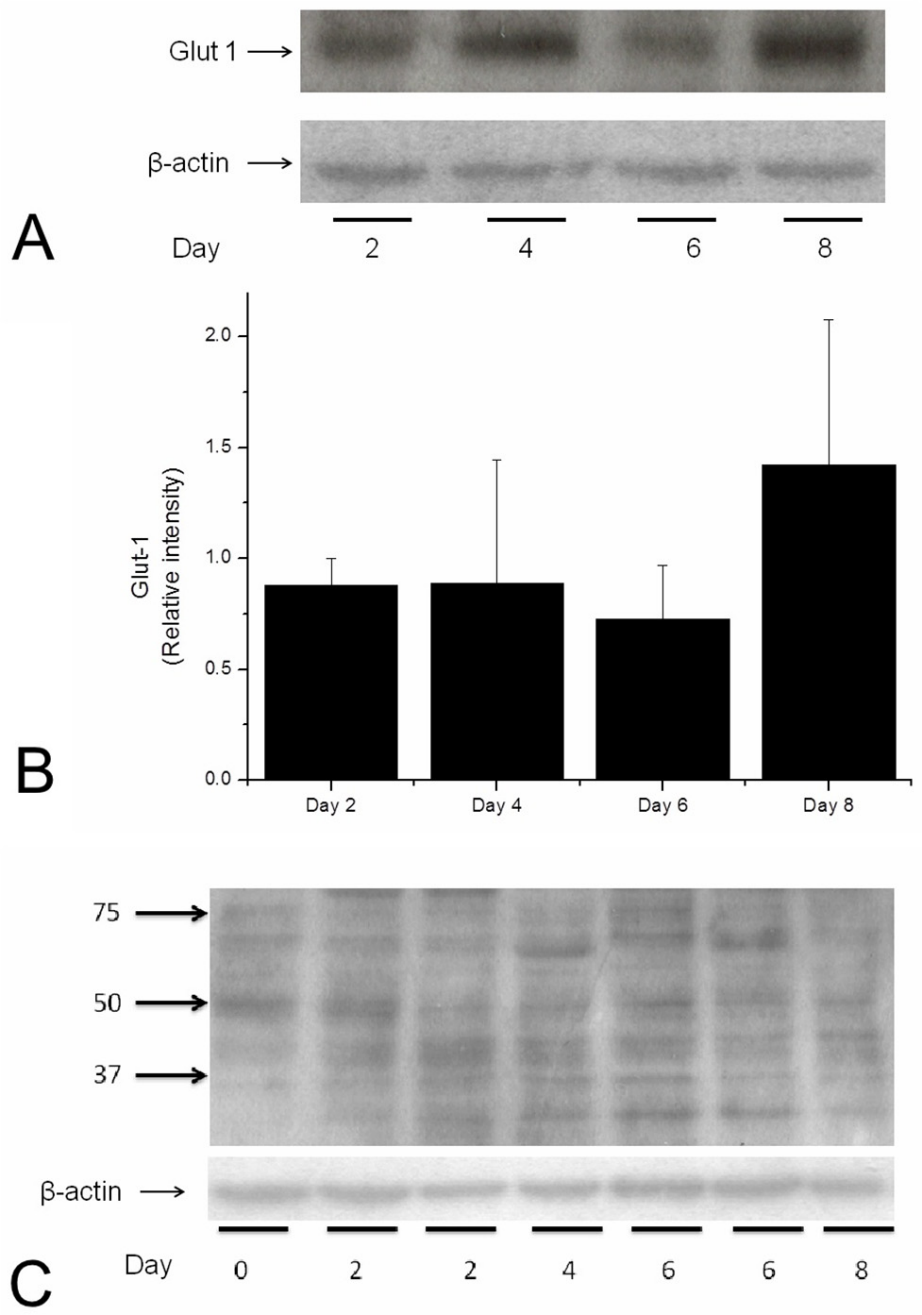
A. A and B. WB of GLUT1 in cultured discs of the prelaminar optic nerve head, which is composed primarily of astrocytes. A) Well-formed, compact bands of GLUT-1 compared to the reference protein (β-actin). B) Quantification bars of two experiments. As before, the number of discs harvested was increased with culture time to compensate for cell loss due to apoptosis. The levels of GLUT-1 are fairly stable, with a small increase at approximately day 8, indicating a more stable culture (see text). The values are the means, with the standard errors being represented by vertical bars. C) A sample WB of GLUT-3 in prelaminar cultured discs of the prelaminar optic nerve. No neat bands are observed, and the predicted GLUT-3 location does not show a clear band. GLUT-3 was detected in the tissue by other means (immunocytochemistry), but the level of the target molecule is insufficient to distinguish signal from noise on WB.

**4. Biochemistry, Western Blot.** GLUT1: Samples from culture days 0 (controls), 2, 4, 6, and 8 were tested, with all of the pieces of the same day being homogenized together. Neat bands for each day were present at the level of the 50 KDa (**Fig 8A**). Quantitative estimates showed uniformity in the presence of the transporter isoform from day 0 to day 6 and an increase on day 8, with no statistically significant differences. (**Fig 8B**).

GLUT3: The same homogenates used for GLUT1 were tested for GLUT3. Despite a positive control band from gray-matter homogenate from rat brain (data not shown, compared to unspecific bands), no neat bands were observed for the prelaminar-tissue homogenates at the expected level of the 54 KDa. Quantitative estimations showed no appreciable amount of the isoform in fresh or cultured tissue. (**Fig 8C**).

### Results of Histological Study

**Fig 9:**
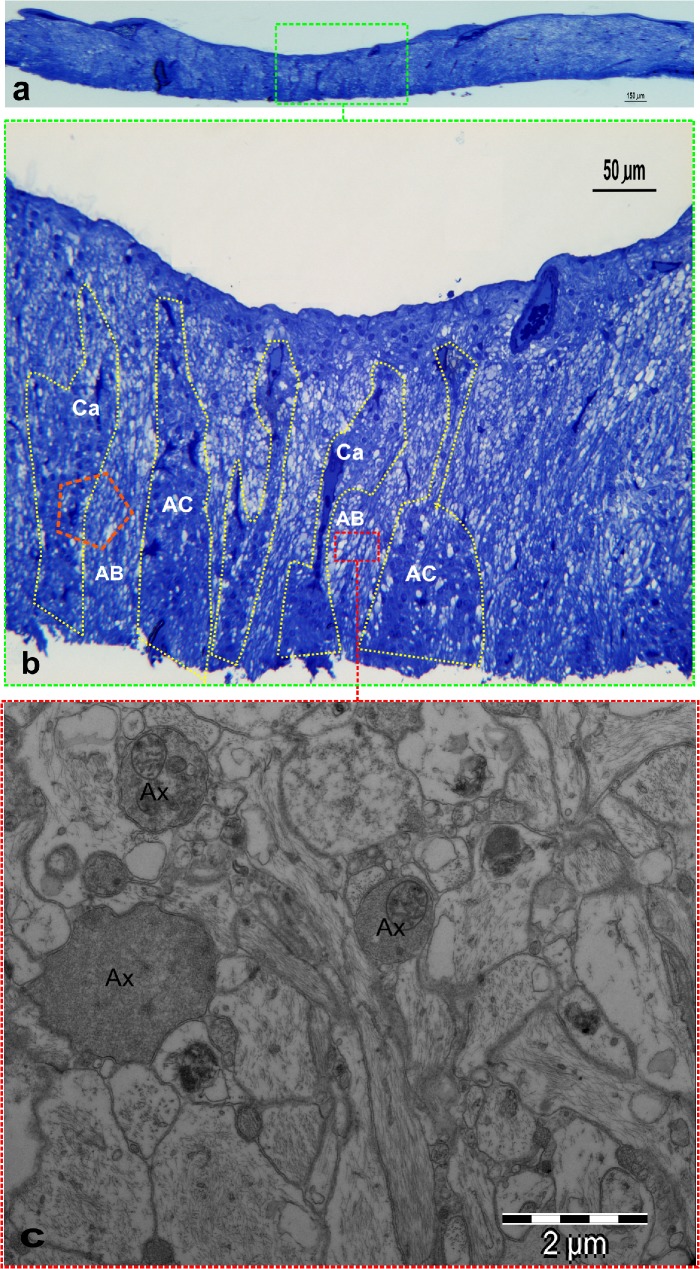
Histology of the prelaminar explant LM and TEM. **a**) Semithin section of a full-length prelaminar explant following excision. The thickness varies between 100 and 300 microns, being thinner in the center. The central thickness in this case is approximately 280 microns. Toluidine blue inclusion, epoxy resin; magnification in the figure. **b**) Central zone (green square) in a). The arrangement in columns of astrocytes is highlighted by the dotted line in part of the section. Axonal bundles pass between the astrocytic columns. The area of the orange pentagon includes a capillary as part of a column and a portion of an axonal bundle. This type of section allows for GLUT-1 labeling in the endothelial vascular cells to serve as a positive control for axonal labeling of GLUT-1. **c**) The area enclosed in the red square is formed exclusively by axons wrapped in an extremely tight web of astrocytic expansions. The complexity of the arrangement is reminiscent of the neuropil in the central nervous system. The TEM micrograph shows three axons (Ax) and numerous astrocytic expansions. This type of section allows for the identification of the involved cells even in the absence of membrane counterstaining, which is required for immunolabeling with gold micelles. Cell identification is aided by the presence of GFAP and Neurofilament staining. The magnification is indicated in the figures.

**Fig 10:**
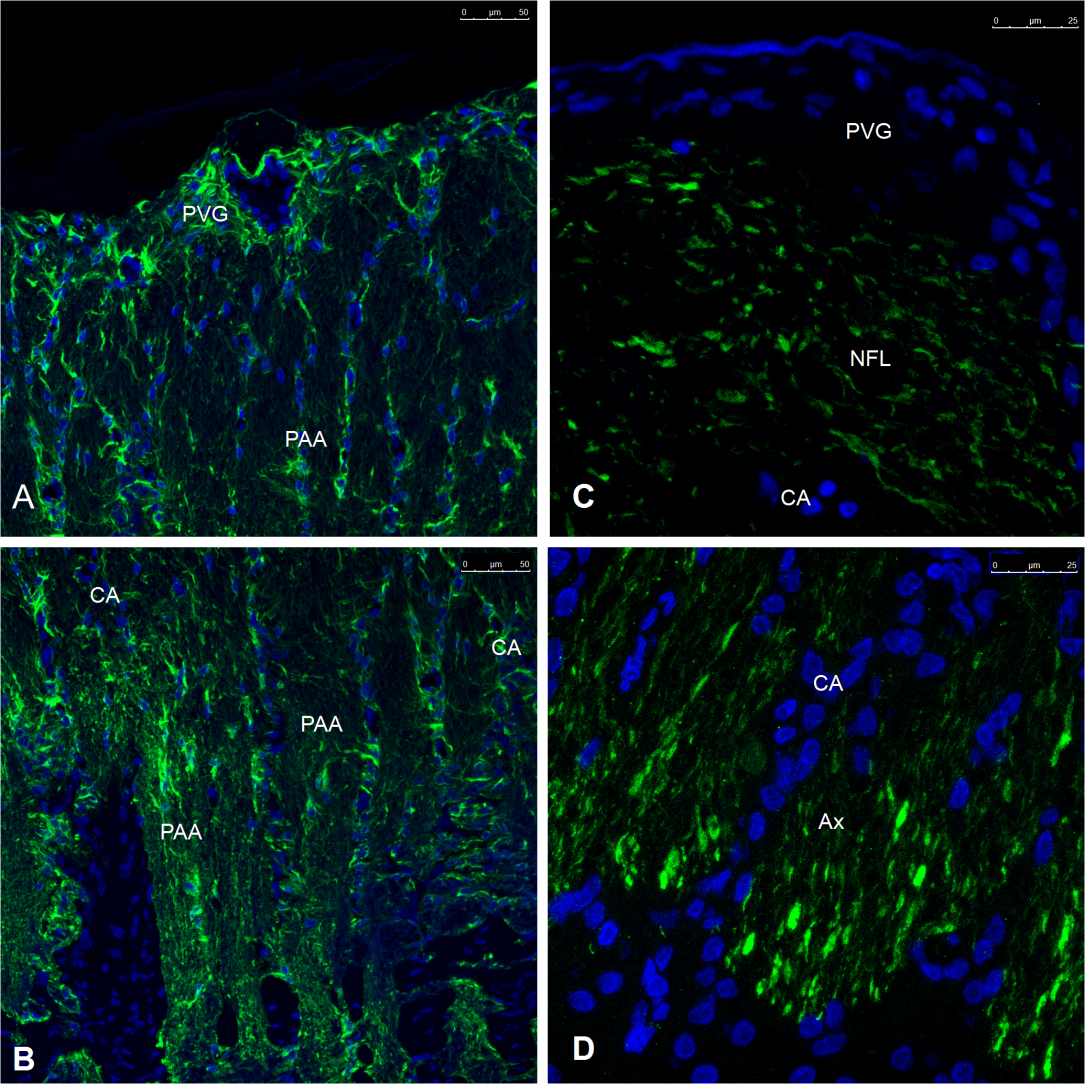
CLSM micrographs of the prelaminar region labeled for Glial Fibrillary Acidic Protein (A, B) and Neurofilament. A, **B**) An axon-free perivascular glial sheet surrounding the trunks of the central vessels can be distinguished (PVG) based on the distribution of the axons in the prelaminar region. The remaining prelaminar tissue contains axons that are wrapped in astrocytic expansions (PAA). The somas of the astrocytes are loosely distributed in the superficial fiber layer and pile up in columns on top of the beams of the *lamina cribrosa* (dark areas at the bottom of **B**). **C, D**) Labeling with neurofilament delimits the superficial fiber layer, with a branch of a central vessel with the unlabeled perivascular glial sheet on top (PVG). The axons form bundles that pass between the astrocytic columns and formed by the piles of astrocytic somas (unstained dark area at the bottom). The magnification is given in the figures.

1. **Morphology with CLSM.** A general view of the architecture of the prelaminar tissue is shown in **Fig 9**. The thickness of the excised explant generally did not surpass 300 microns on average. The tissue structure of the fiber layers of the prelaminar ONH was intricate (**Fig 9C**). Numerous thin astrocyte projections intermingled with axons and other astrocyte projections, forming a mesh that defied the resolution power of fluorescent confocal laser microscopy. The two main cellular elements in the tissue are astrocytes, as manifested by the presence of strong GFAP staining (**Fig 10A and 10B**), and the axons of the retinal ganglion cells (RGCs), as seen out by Neurofilament (NF) staining (**Fig 10C and 10D**).

**Fig 11:**
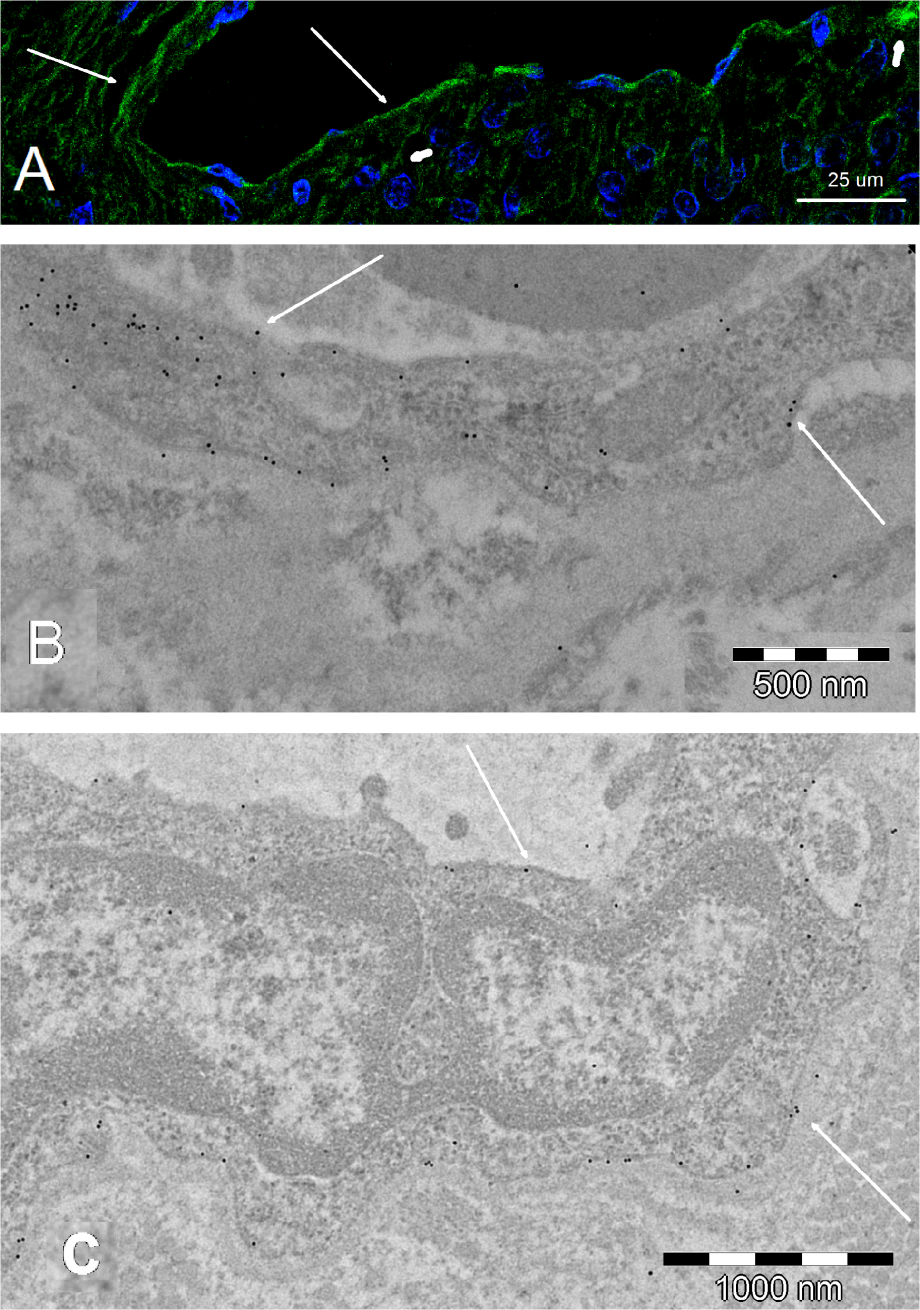
A: CLSM; B and C: TEM. All of the micrographs show GLUT1 labeling (gold micelles, arrows) in the adluminal and abluminal membranes of the vascular endothelial cell. Thick arrows in **A** point to abluminal staining as detected using CLSM. This is a key figure because TEM ultrathin sections included both vascular endothelium- and astrocyte-wrapped axons. In addition, the precise lining of the GLUT1 labeling in the vascular endothelium acts a s a positive control for labeling in the axonal membrane, as seen in Fig 11. The contrast between the endothelium and its surroundings allow for easy localization of the otherwise invisible plasmatic membrane. The magnification is given in the figures.

GLUT1 was detected at the membranes of the vascular endothelial cells in the capillary network of the central artery and vein of the retina. Gold micelles neatly delineated the abluminal and adluminal membranes of the endothelial cells (**Fig 11A, 11B and 11C**). GLUT1 was also detected in (1) the end-feet of the astrocytes of the fiber layer; (2) in the prelaminar region, where the somas were stacked, forming astrocyte columns; and (3) where capillaries were located (Fig **11A, 11B and 11C**).

**Fig 12:**
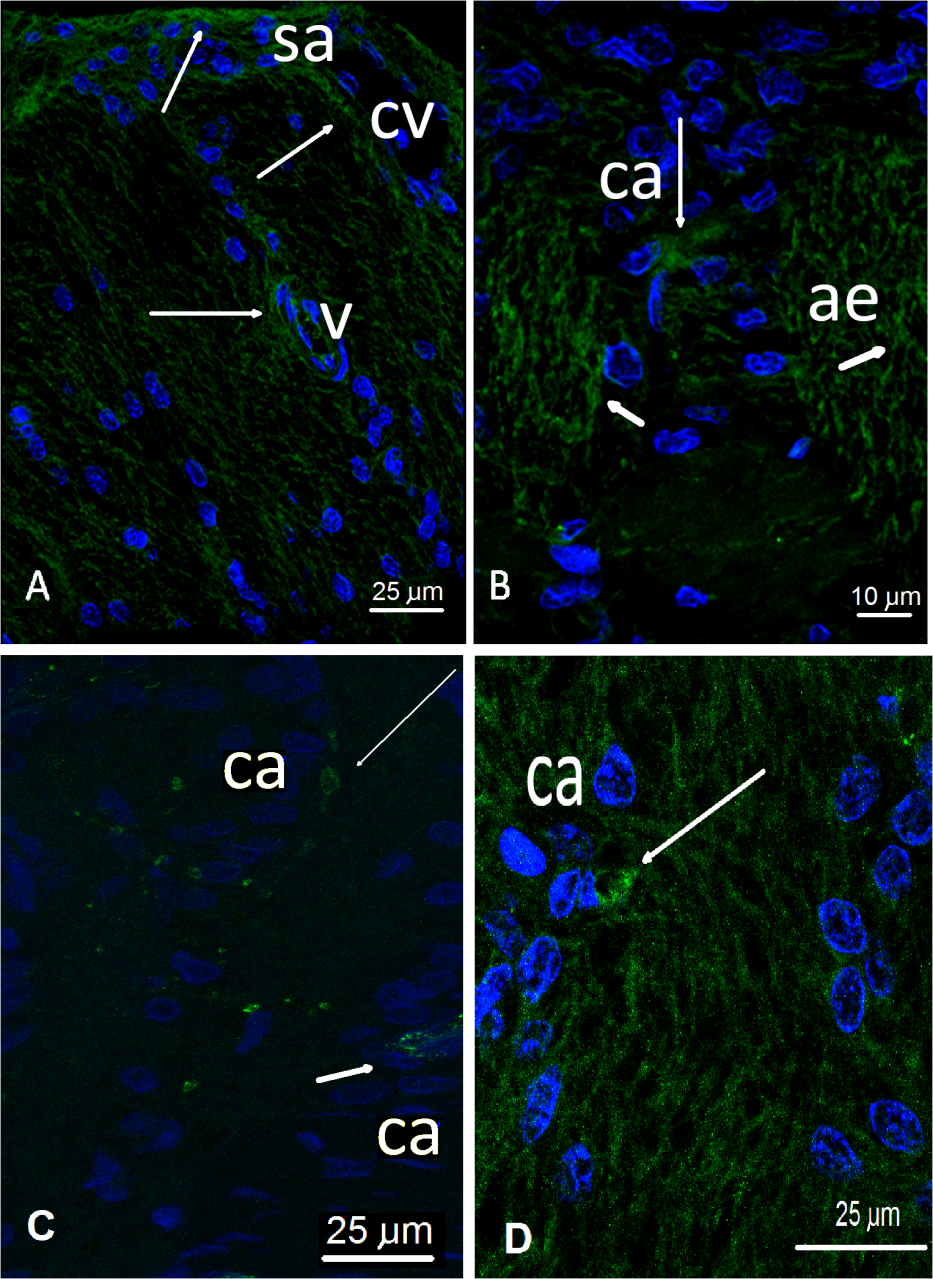
CLSM. Astrocyte nuclei are shown in blue (DAPI). Positive staining of the target (GLUT1) shows green fluorescence. **A, B**: GLUT1, long thin arrows point to positive green fluorescence in the surface astrocytes (sa) near the central vessels (cv), the astrocytes surrounding the capillaries (v), as well as in the columnar astrocytes (ca). The short thick arrows point towards their axonal expansions (ae). **C** Specific staining of GLUT-1 at the level of the columnar astrocytes (ca, short arrow) as well as in the axonal bundles (long arrow). **D** GLUT1 labeled by a chain with a secondary anti-idiotypic antibody. The long arrow points towards an astrocytic expansion that enters into the axonal bundle.

GLUT1 was detected at the membranes of the perivascular astrocytes surrounding the branches of the central artery and vein of the retina. These astrocytes lie primarily on the surface of the optic disc, forming an incomplete Elschnig’s membrane(**Fig 12A, sa**). Additionally, GLUT1 was detected in the axonal bundles at the membranes within the axonal expansions of the astrocytes wrapping RGC axons. In contrast to the retinal nerve-fiber layer, the axonal bundles were densely packed and no capillaries were detected (**Fig 12A, 12B, 12C and 12D**).

**Fig 13:**
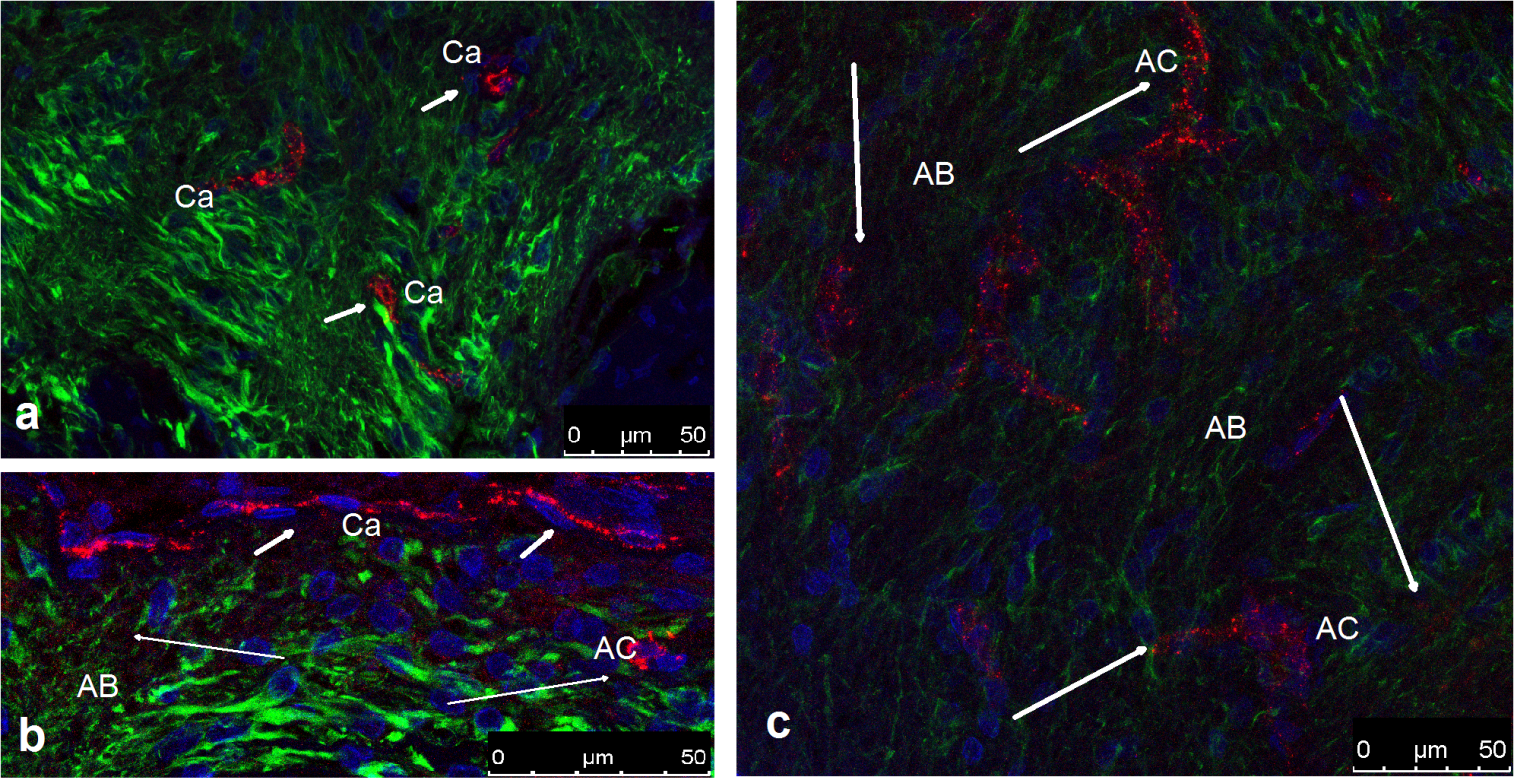
CLSM. The colocalization of GLUT-1 and GFAP in the axonal bundles and astrocytic columns (**a, c**) and in the nerve fiber layer (**b**). The nuclei are stained in blue (DAPI), GFAP appears in green, and GLUT-1 in red. **a**) When GFAP staining is intense, only the vessels (capillaries) show intense red staining in the endothelium, indicating the abundance of GLUT-1. b) A capillary running across the nerve fiber layer shows intense GLUT-1 labeling (short arrows). However, the presence of specific GLUT-1 labeling is also discernible (long thin arrows) in the somas of the astrocytes (AC) and less intensely at the beginning of the axonal bundles (AB). **c**) Diminishing the signal from GFAP allows the specific staining (thin arrows) of GLUT-1 to be seen both at the level of the somas (AC) and bundles (AB).

Double labeling with GLUT1 and GFA unequivocally showed that astrocytes bear the glucose transporter both on the somas and on perivascular end-feet, as in the periaxonal expansions in the axonal bundles. (**Fig 13A, 13B and 13C**).

**Fig 14:**
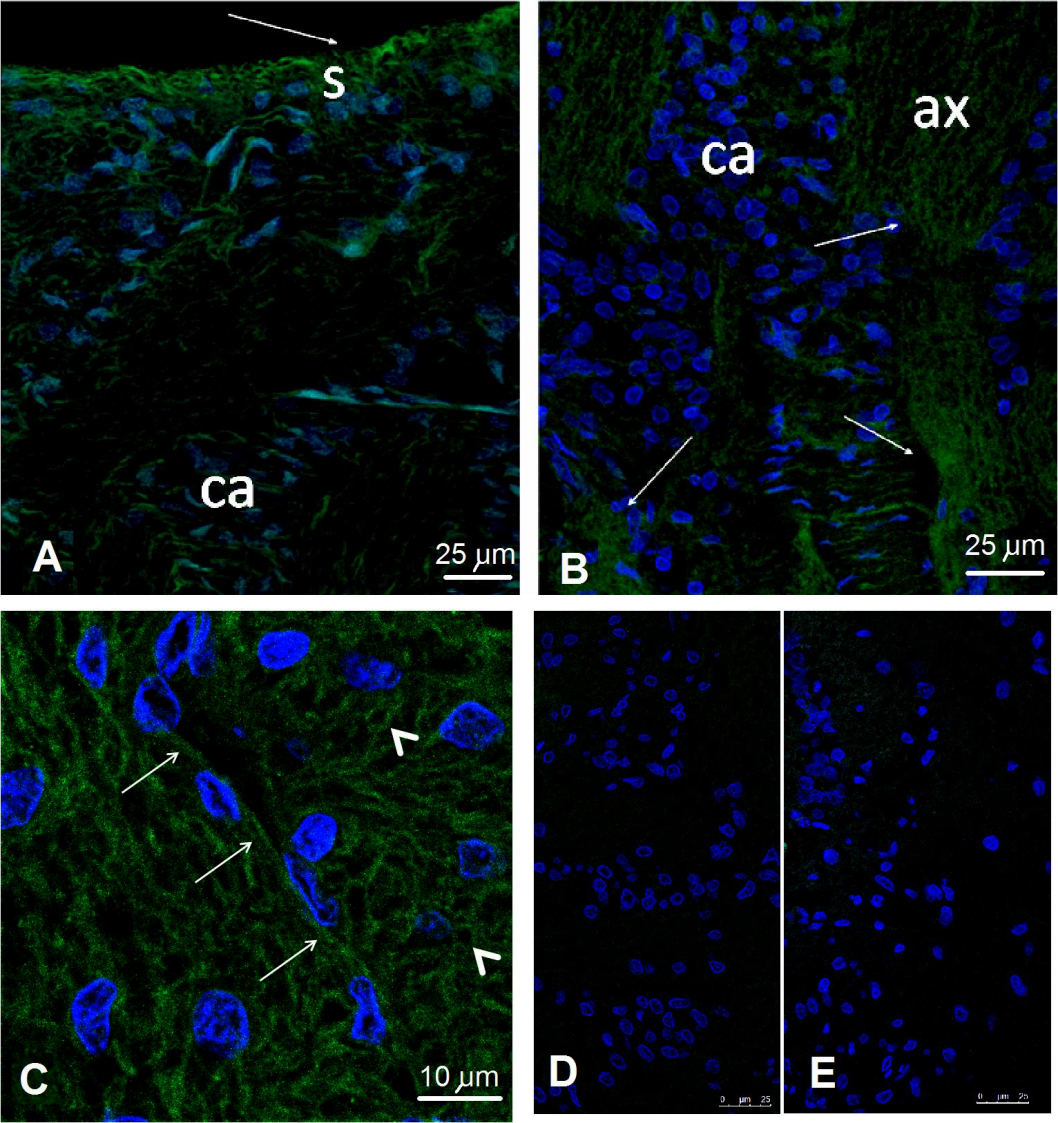
CLSM. The astrocyte nuclei are shown in blue (DAPI), with positive staining of the target (GLUT3) being shown in green fluorescence. **A**, **B**: Long thing arrows point towards positive GLUT3 staining in the surface-fiber layer, away from the vessels (s) and axon bundles (ax). **C**) GLUT3 is labeled by a chain with a secondary anti-idiotypic antibody, showing the staining of axons cut longitudinally (medium size arrows) and transversally (arrowheads). D) Idiotypic control for GLUT-1 (Fig 12D), E) Idiotypic control for GLUT-3.

GLUT3 was detected in the membranes of the axons in the neural-fiber layer of the ON, forming the more superficial layer of the nerve where the axons bend towards the *lamina cribrosa*. Staining was also observed in the more deeply situated axonal bundles of the prelaminar region on top of the *lamina cribrosa* (**Fig 14A, 14B and 14C**).

**Fig 15:**
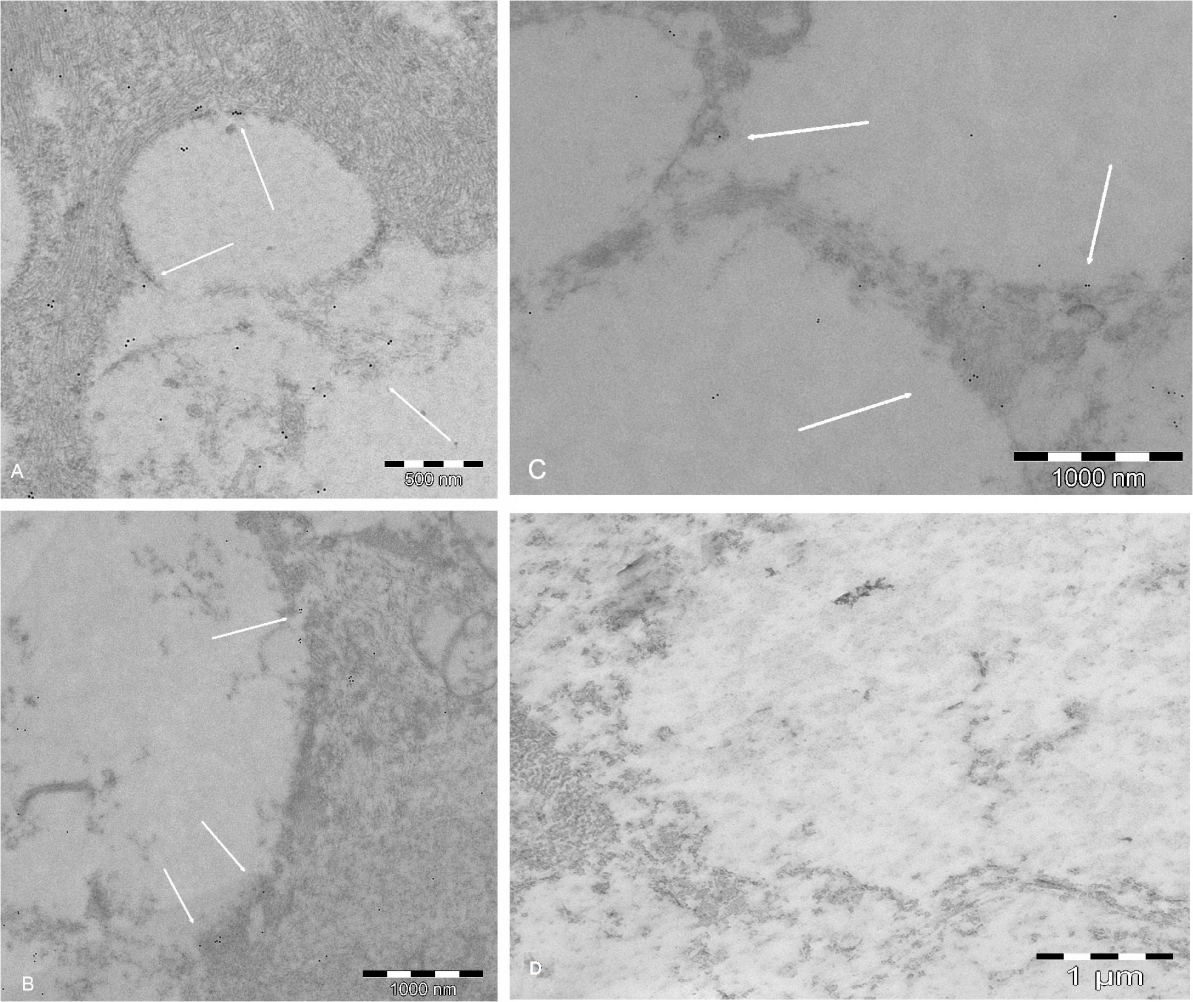
TEM micrographs. **A**, **B** and **C**: GLUT1 colloidal gold labeling in the axonal bundles. The gold micelles (arrows) delimit the boundaries between the clear areas (axons) and the GFAP-rich darker areas of astrocytes. Notably, this area is in the same ultrathin section in Fig 9, which shows the specific distribution of the labeling in the endothelial vascular cell. **D**) Control without primary antibody. The magnification is given in the figures.

**Fig 16:**
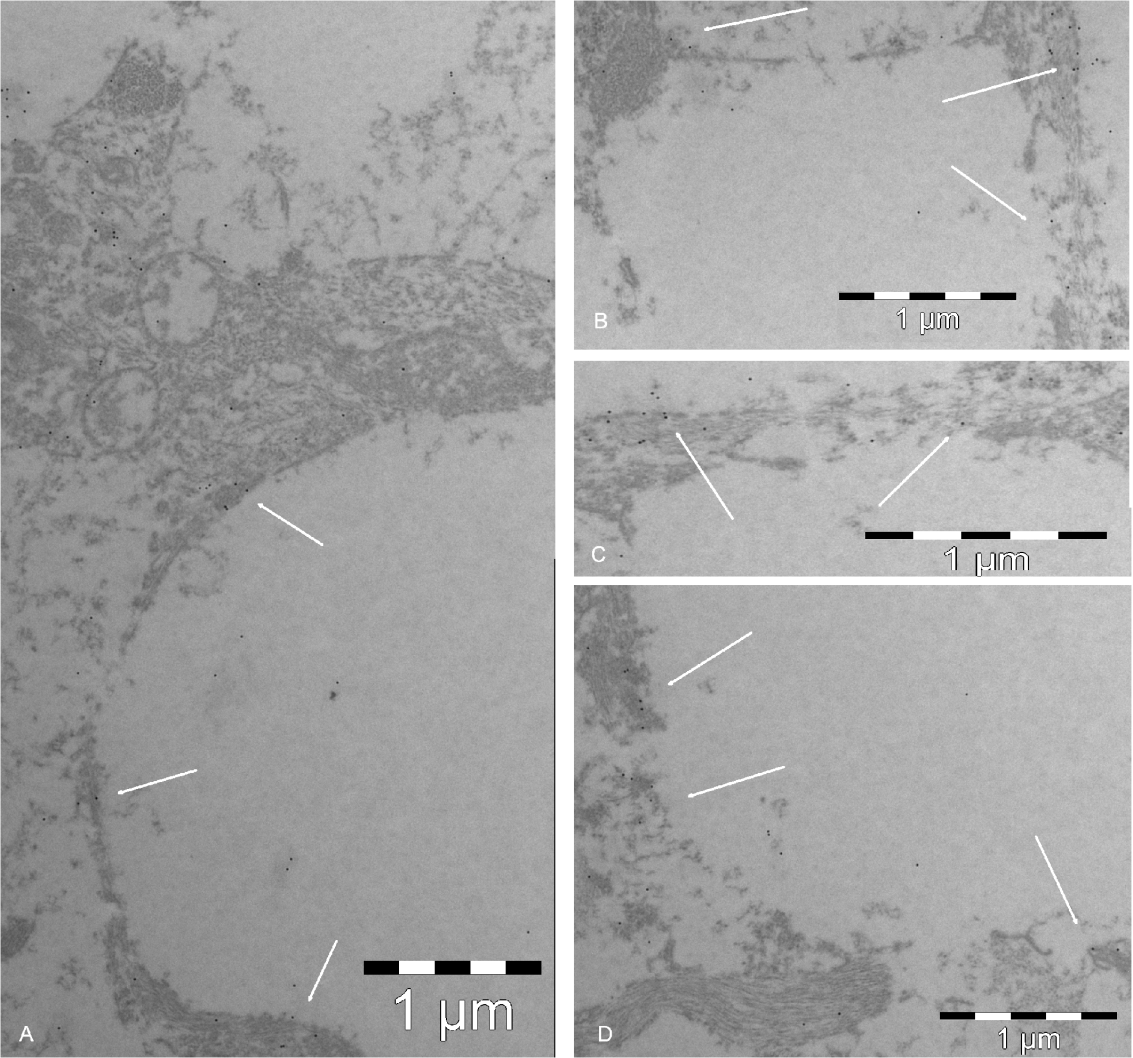
TEM micrographs. **A**, **B**, **C** and **D**: Colloidal gold immunolabeling of GLUT3. The gold particles (arrows) delimit the boundaries between the clear areas (axons) and the GFAP-rich darker areas of astrocytes. The magnification is given in the figures.

**2. Morphology with TEM.** The lack of ultrastructural detail on TEM (**Figs 11**, **15** and **16**), most notably the absence of apparent membranous structures, is a consequence of the correct use TEM immunolabeling techniques. As detailed in the Methods, the use of contrast with heavy metals is omitted to avoid confusion with the gold micelles. The price of gold visibility is high in that the plasmatic membrane and the organelles, although present in the section, are not visible. Nevertheless, the axon bundles, in which axons are surrounded by astrocytic cytoplasmic expansions, can be discerned. These remain visible (1) because the area was selected based on the LM study of semithin sections, and (2) because GFAP-rich areas in astrocytes can be distinguished from the clearer neurofilament areas in axons. The gold mark is fairly distributed along the boundaries, corroborating the specificity of the labeling, the vascular endothelial cells, present in the same ultrathin section, show a precise lining with gold micelles, clearly indicating that the target is GLUT1.

**Fig 17:**
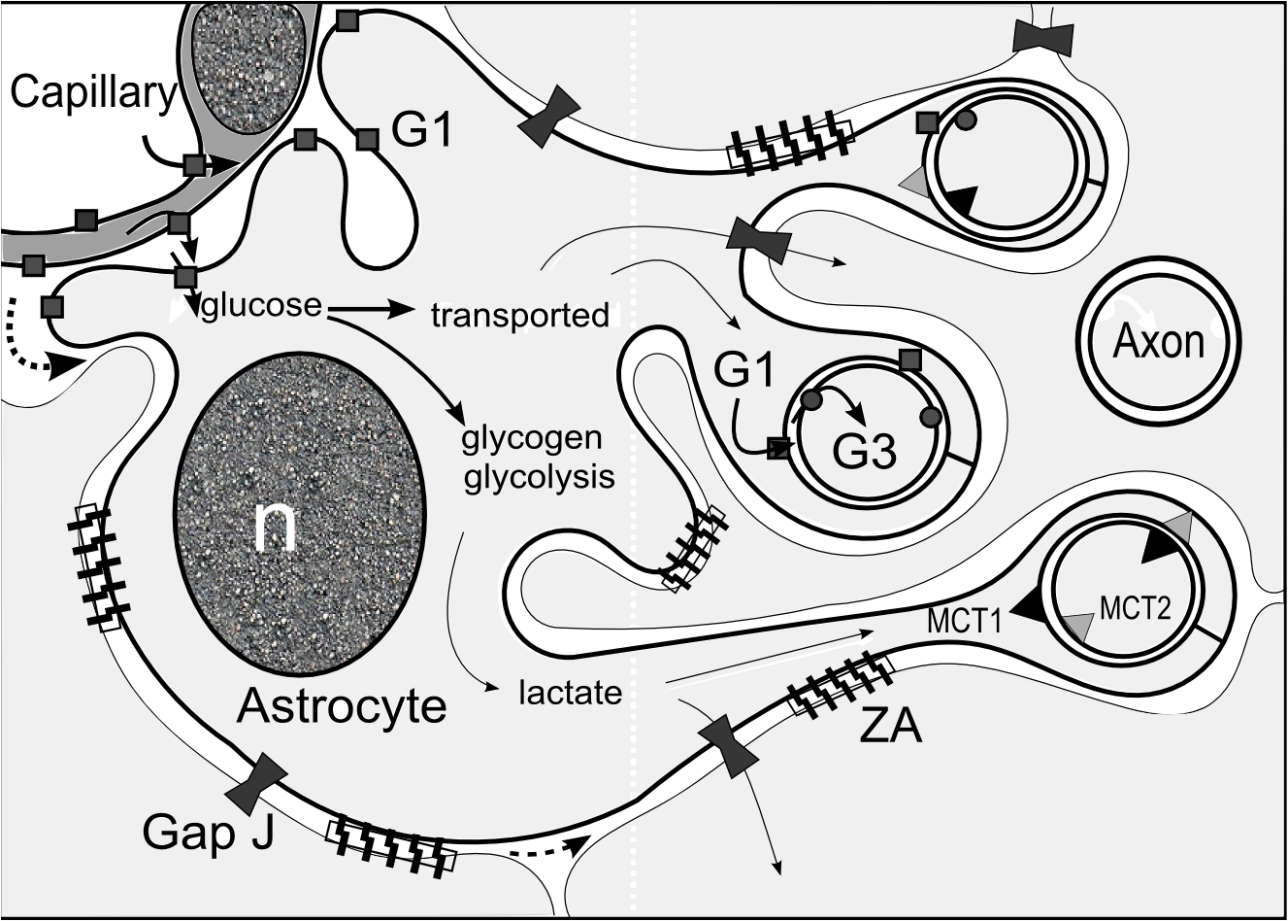
Diagram illustrating the main molecular complexes for the transport of glucose and lactate in the prelaminar tissue. GLUT1 transporters (G1) are present in the adluminal and abluminal membranes of the capillaries in the astrocyte columns. Glucose is transported passively from the capillary lumen into the interstice. On the other side of the capillary basal membrane (not represented), the end-feet of the astrocytes also express GLUT1 transporters, which bring glucose into the astrocyte cytoplasm. Once in the cytoplasm, glucose may pass to other astrocytes through gap junctions, be stored as glycogen, or undergo glycolysis to render lactic acid. Glucose is also transported to the axonal cleft by GLUT1 itself. From the axonal cleft, glucose is introduced into the astrocyte by the GLU3 transporter (G3). Similar to glucose, lactate can pass freely to other astrocytes through gap junctions (Gap J) and be delivered to the axonal cleft by means of monocarboxylate transporter MCT1. Lactate is introduced into the axon by means of MCT2. Axons also express MCT1 on the membrane, conferring the ability to eliminate excess lactate and other monocarboxylates, which can be recovered by astrocytes that also possess MCT2. The text “ZA” indicates calcium-dependent zonulae adherens, N-cadherin mediated complexes that help maintain tissue integrity. n: nucleus of the astrocyte. (Dark square,G1: GLUT1; dark circle, G3: GLUT3; dark triangle: MCT1; light triangle: MCT2; dark hourglass, Gap J: gap junction; zipper, ZA: *zonula adherens*; n: nucleus).

GLUT3, which is not present in the vessels, follows the same pattern as GLUT1 in the bundles. Both transporters line the intercellular cleft for the transport of glucose from the astrocyte into the axons. As the final graphic abstract shows (**Fig 17**), both glucose and lactate are transported in the prelaminar tissue (which is not the case in other areas of the optic nerve), and this system depends on the maintenance of narrow intercellular clefts, as shown by the presence of zonula adherens.

Ultrathin sections prepared for immunolabeling typically do not show any cell membrane, and the position of the membrane must be deduced from the presence or absence of other cytoplasmic features, such as intermediate filaments (e.g., GFAP) for astrocytes and neurofilaments for neurons. GLUT1 was detected at the perivascular expansions of the astrocytes in the astrocyte columns. In the same ultrathin sections that include specifically labeled vascular endothelium, GLUT1 antibodies labeled the astrocyte boundaries in the axonal expansions that interleaved the axonal bundles (**Fig 15A, 15B and 15C**).

GLUT3 was detected at the boundaries of the axons in the axonal bundles (**Fig 16A, 16B and 16C**). The qualitative estimation of the micrographs rendered a similar staining pattern in the axonal bundles for GLUT1 and GLUT3, suggesting a similar amount of both transporters on both sides of the intercellular cleft.

## Discussion

### Tissue culture

Tissue culture of the prelaminar region of the optic nerve of the domestic pig can be used as a model to study the response of astrocytes to environmental changes. For this method, it is necessary to know when a culture stabilizes sufficiently to allow experiments to be performed. Characterization of the culture implies knowledge of the main metabolic pathways that govern the maintenance of the tissue conditions to mimic the physiological state. Our *in toto* culture is not properly organotypic in the sense that the axons of the retinal-ganglion cells are unavoidably cut. The axons thus decay and vanish from the culture. The advantage of *in toto* culturing is that astrocytes preserve their tissue structure and relationships, including gap junctions and the zonulae adherens (**Fig 2D**). Those relationships are very difficult to induce in monolayers of cultured astrocytes.

However, one possible drawback of *in toto* tissue culture of the prelaminar region is that different morphological techniques, including CLSM, are not as neatly performed as in monolayers. This difficulty arises because astrocyte contacts are intricate, and dye penetration is incomplete. This occurrence is apparent in our results, both with JC-1 and TUNEL. Axons depend on astrocytes, but not vice versa^3^, and astrocyte culture can be maintained for a number of days after axons disintegrate, which occurs during the first 24 h of culture. Our results with decreasing amounts of LDH in the culture medium suggest that necrosis occurs in the explants, likely as a consequence of the sectioning and manipulation of the tissue during the explanting procedures. LDH levels are reduced and seem to stabilize around day 6. From day 6 onwards, cell death by necrosis can be considered negligible.

Increasing levels of caspase 7 in the first days of culture indicate that apoptosis takes place and increases during the first few days. The presence of apoptosis in the culture was examined by TUNEL and JC-1 staining. In our experiment, both procedures showed a high degree of variability in relation to penetration into the tissue. In contrast to cell-monolayer cultures, tissue culture preserves relatively thick masses of tissue. The dye penetration under such conditions is random and can be limited, and the absence of staining either with TUNEL and JC-1 does not preclude the presence of apoptosis. This potential for false-negative results is shown by images of altered nuclei that display karyopyknosis and karyorrhexis with DAPI staining but that are negative for TUNEL or JC-1 staining. Positive TUNEL and JC-1 staining nevertheless confirm that apoptosis truly takes place in the stained cell (**Figs 4 and 5**). Caspase activation, which occurs in the mitochondrial apoptosis pathway, is linked to mitochondrial outer membrane permeabilization (MOMP). [33] MOMP leads to cell death because of the release of caspase-activating molecules and metabolic failure in the mitochondria. [34] Mitochondrial metabolic failure can be visualized by JC-1 staining (**Fig 5**).

Our results with caspase 7, the levels of which were quantitatively measured, showed that the cultures tended to stabilize from day 4 onwards. At approximately day 8, the apoptosis levels increased again to levels observed on day 2 (**Fig 7**). Both CLSM and TEM revealed that some areas of the explants underwent no morphological changes. In these areas, the nuclei appeared healthy under DAPI staining, the zonulae adherens were clearly visible (not shown), and capillary-astrocyte relationships were maintained. These characteristics can be seen in **Fig 2**, in which the endothelium, basal membrane, and astrocyte end-feet are well preserved. This result reinforces previous data indicating that cultures of the prelaminar tissue continued up to day 12. [20] Previous assays had shown that at approximately day 8, there was sufficient remaining tissue to be experimentally used. Here, we confirm that apoptosis levels at day 8 were low, and sufficient tissue was preserved for conducting apoptosis induction assays. Additionally, levels of GLUT1 were maintained in tissue culture without significant variation up to the day 6 of culture, with levels increasing on day 8 (Fig **8B**). This observation can be interpreted as an additional clue to the stabilization of the culture at approximately day 8. The persistence of GLUT1 in the prelaminar region of the cultured tissue is a mark of culture stability and supports its use in experiments of astrocyte anoikis as a model for glaucoma pathogenesis.

### GLUT Expression

To be certain that prelaminar axons take up glucose from the intercellular cleft, which is delimited by axonal and astrocyte membranes, we must meet two requirements: (1) GLUT1 must localize on the astrocytic side of the cleft in fresh tissue and (2) GLUT3 must localize on the axonal membrane.

The presence of GLUT receptors in neurons (axons) that make up the optic nerve can be interpreted from the perspective of the debate over the relative role of glucose and lactate as the primary energetic resource of neuronal metabolism in the central nervous system. It has been reported that the GLUT3 protein is strongly expressed in gray-matter regions of the brain but only weakly expressed in white matter. [35] Our CLSM and TEM results point towards a very specific localization for GLUT3 in the membrane of the axons (**Fig 12**), together with a precise localization of GLUT1 in the membrane of the astrocyte soma, the vascular end-feet and the processes that wrap axons (**Fig 8**). Our TEM ultrathin sections were large enough to include the columns of astrocytes and the axonal bundles in the same section. The sections that also included capillaries were selected for anti-GLUT1 gold immunolabeling. In this way, the vascular endothelium was used as a positive control as GLUT1 was found to be present in both abluminal and adluminal endothelial membranes. Our results show a delineation of the endothelial membrane with gold micelles, indicating specific labeling. In the same sections, positive immunolabeling of the axonal bundles indicate the presence of GLUT1 in the astrocyte processes that envelop the axons. More important is the location of GLUT1. Using TEM, GLUT1 was observed in the membranes of the axons within the bundles of the prelaminar region, strongly supporting the idea of the intracellular transport of glucose by the astrocyte to the axonal cleft. Like most cells, astrocytes take up glucose from interstitial fluid via a passive transport process. In contrast to the sodium-glucose symporter, which can transport glucose from low to high concentrations and is found in the enterocytes and the proximal tubule of the nephron [21], GLUT transporters facilitate the movement of glucose across the plasma membrane using the difference in glucose concentrations. [36] This result indicates that GLUT1 in the astrocyte membrane, far from the capillary, can transport glucose beyond the cytoplasm into the axonal cleft, not the opposite. This conclusion can be reached because the glucose concentration in the cleft is expected to be lower than in the astrocyte cytoplasm given that glucose is removed from the cleft by the axon.

Contrary to our expectations, western blotting of GLUT3 failed to distinguish signal from noise in homogenates of fresh prelaminar tissue (**Fig 8C**). Consequently, we could not take advantage of the disappearance of the axonal fragments to quantitatively measure the reduction of the associated GLUT3. Other studies have failed to localize GLUT3, a neuronal transporter of glucose, in gray matter. GLUT3 has been located primarily in pre- and postsynaptic nerve endings and in small neuronal processes. [26] Immunohistochemical [37] and electron microscopic studies [26] have illustrated that GLUT3 is localized in the neuropil, is largely absent from neuronal cell bodies, and is absent from white matter. [23] In our tissue, the GLUT3 probe resulted in no signal on a western blot and positive TEM labeling. A clear western blot result for GLUT1 together with a negative result for GLUT3 can be interpreted as positive in the sense that it indicates the absence of a cross reaction in the antigenicity of the antibodies used. This fact strengthens the specificity of the detection of the morphological studies given that the cross reaction of antibodies can decrease the reliability of findings of the molecule being searched for in the tissue.

Three points should be considered with respect to the relative number of GLT and MCT transporters:

1. GLUT1 is present both in vascular endothelial cells and astrocytes. In astrocytes, GLUT1 transporters are present both in the capillary end-feet and in the axon-wrapping expansions. GLUT3 is present only in axons. The relative amount of GLUT1 must be therefore be greater.
2. As is observed in the white matter of the brain [35], compared to GLUT1, GLUT3 expression is very scant in the prelaminar region. However, the transport of glucose is more rapid in the case of GLUT3 compared to GLUT1. Although GLUT1 and -3 are both high-affinity glucose transporters, GLUT3 has a higher glucose affinity. [38] As a result, a lesser amount of GLUT3 is needed in the axonal side of the cleft compared to the astrocytic side (GLUT1) for the same transportation load. Our TEM images of axonal bundles show similar labeling for GLUT1 and GLUT3 based on qualitative estimations. This result is compatible with a coupled activity of the transport of glucose to the intercellular cleft and intake by the axon.
3. The similar antibody detection of the two GLUT isoforms by immunolabeling in the TEM preparations suggests that the low tissue concentration of GLUT3, located only in the axonal bundles, falls below the sensitivity threshold of WB. However, GLUT3 can be precisely located using the much more sensitive immunolabeling procedure. A negative western blot result for GLUT3 simply indicates that the level of the target is below the sensitivity of the test and that the signal cannot be separated from the noise. Although the specificities of TEM immunolabeling and western blotting can be identical if the same primary antibody is used, the sensitivity of TEM is much higher because it is capable of detecting and labeling individual molecules.

### Blood-brain barrier (BBB) and the prelaminar tissue

It has been reported that GLUT1 expression at the blood-brain barrier (BBB) is influenced by astrocytes and other glial cells. Then, the brain needs more glucose, GLUT1 expression in the brain endothelial cells (BECs) becomes upregulated. [39,27] It is known that rat capillary endothelial cells in primary culture often express GLUT3, a transporter that is not present in capillaries *in vivo*, as a result of cell de-differentiation in culture. [39] In our study, TEM detection of GLUT isoforms has been conducted under normal tissue conditions, but differences in the expression of membrane glucose transporters are possible during physiological changes, such as exercise [40], stress or pathological conditions.

The BBB resides at the endothelial level in the tight junctions between two adjacent cells. It is formed essentially of transmembrane proteins, including occludin, claudins, and junctional adhesion molecules (JAM). [27] Once in the extravascular space, glucose can reach the axonal intercellular cleft in two ways, extracellularly and intracellularly. Unlike epithelial-type cells, astrocytes do not form impermeable barriers. The BBB acts as a selective interface that insulates the CNS parenchyma from the circulation. [41] This barrier has its histological basis in the complex tight junctions of microvascular endothelial cells (MVECs), restricting permeability by means of claudins and occludin. [42] Astrocytes contribute to the maintenance and repair of the endothelial barrier, but astrocyte unions are focalized. Connections between astrocytes do not form continuous barriers, and fluid can circulate between them. [43] Membrane apposition by means of individual molecules [44] is the main cell-attachment mechanism in the vitreous interface, and, along with gap junctions, the *zonulae adherens* is the only attachment junction among astrocytes. [45] This cell architecture of the prelaminar region enables the unimpeded passage of fluids from the vitreous cavity due to the complete absence of tight junctions, as has been shown by perfusing the vitreous at high physiological pressure. [46,47]

Similarly, fluid and solutes that extravasate from the capillaries could reach the axonal bundles if there were a sufficient perfusion pressure gradient (**Fig 17, dotted arrows**). Despite the reduced space available, the tortuosity of the intercellular cleft and the long distance between capillaries and axons, extravascular fluid and solutes, including glucose, can reach the axonal bundles. However, in the absence of edema, the glucose that reaches the axons must be insignificant. Glucose is incorporated into astrocytes at the vascular expansions but, due to the reduced space available at the intercellular cleft, the diffusion of glucose in the extracellular space is probably insufficient to nurture the axons. The presence of GLUT1 on the astrocytic side of the axonal cleft, together with the high ratio of intracytoplasmic to extracellular volume in the prelaminar tissue, implies the astrocyte transports intracellular glucose to make it available in the axonal cleft. The presence of GLUT1 on the axonal face of the astrocyte expansions suggest that glucose is transported to the axonal cleft intracytoplasmically and delivered to the cleft by the GLUT1 transporters on the astrocyte membrane. This model is compatible with the concept of an astrocyte syncytium for intracellular metabolite trafficking. [19] Astrocytes are connected to each other at gap junctions that can allow small molecules (MW<1,000) to be exchanged [12], including glucose. [48] There is therefore no need for all astrocytes that wrap axons to have processes that make contact with capillaries. In the retina, naked axons form tight bundles protected by Müller cells, with the very scarce presence of astrocytes [17]. In contrast, the astrocyte-rich prelaminar region maintains tissue integrity thanks to the abundance of the calcium-dependent zonulae adherens. These junctions help to anchor the cell between them and form part of a wider signaling system, the breakdown of which may trigger anoikis in astrocytes. [20] As monocarboxylate transporters have been described in the prelaminar region of the ONH, this transitional area is likely to use both lactate and glucose, which would make it more resistant to metabolic stress (these conclusions are summarized in **Fig 17**). Although, due to the scant presence of GLUT3 compared with GLUT1, as shown by western blot, glucose uptake may only be marginal. However, uptake could be significant in abnormal or experimental circumstances. A double-blind randomized study proved that elevated interstitial glucose could improve glaucoma outcomes. [49] Additionally, the Ocular Hypertension Treatment Study (OHTS) found the intriguing result that diabetes could offer some protection against glaucoma. However, after combining the data from OHTS and that of the European Glaucoma Prevention Study (EGPS), the prevalent opinion is that diabetes, on the whole, is not a significant protective factor. [50]

An intriguing hypothesis is that astrocytes in the prelaminar region may induce the expression of MCT transporters in axons, making lactate available to the axon as a significant energy source. In the retina, astrocytes are limited to the nerve-fiber layer (NFL) are scarce in number. Moreover, astrocytes do not wrap RGC axons, which run naked in the NFL. Retinal metabolism is based only on GLUT receptors for glucose. Thus, as found here, both MCT and GLT transporters are present in the astrocyte-rich prelaminar region. As soon as the retrolaminar portion is reached, where the white-matter tract begins and is dominated by myelin-wrapping oligodendrocytes, GLUT transporters are lacking. Lastly, in the neuropil, where neurons alternate with astrocytes, glucose and lactate are again shared as energy sources. As the astrocyte is a source of lactate in the CNS, neurons must express MCT in the membrane for the lactate shuttle to be operative. That astrocytes are able to induce the upregulation of MCT receptors in the prelaminar region is an intriguing hypothesis.

## Conclusions

Given that necrosis only occurs following the initiation of our culture protocol, the main process for cell death under our culture conditions is apoptosis. Apoptotic processes also tend to diminish over time, and the tissue tends to stabilize, making it a suitable tool for apoptosis-induction experiments. The GLUT1 receptor is present in astrocytes, together with endothelial cells, while the GLUT3 isoform is found only in axons. The presence of GLUT1 in the axonal face of the astrocyte expansions suggests that, in the prelaminar region of the ONH, glucose is transported to the axonal cleft intracytoplasmically and delivered to the cleft by GLUT1 transporters in the astrocyte membrane. As monocarboxylate transporters have previously been reported in this transitional region of the optic nerve, this area is likely to use both types of fuel, i.e., lactate and glucose, although the latter is secondary and vestigial. This system of coupled transporters is related to a narrow intercellular cleft, the maintenance of which depends on cell-to-cell contact molecules, of which *zonulae adherens* are feature elements. Separation of the astrocyte by interference with *zonula adherens* junctions would directly compromise energy transport into the axons.
